# Threats and Mitigation Strategies for Electroencephalography-Based Person Authentication

**DOI:** 10.1155/ijta/3946740

**Published:** 2025-02-03

**Authors:** Zaib Unnisa, Asadullah Tariq, Irfan Ud Din, Danish Shehzad, Mohamed Adel Serhani, Abdelkader N. Belkacem, Nadeem Sarwar

**Affiliations:** ^1^Department of Computer Science, Superior University, Lahore, Pakistan; ^2^College of Information Technology, United Arab Emirates University, Al Ain, Abu Dhabi, UAE; ^3^Department of Computer Science, New Uzbekistan University, Tashkent, Uzbekistan; ^4^Department of Computer Science, National University of Computer and Emerging Sciences, Islamabad, Pakistan; ^5^College of Computing and Informatics, University of Sharjah, Sharjah, UAE; ^6^Department of Computer Science, Bahria University Lahore Campus, Lahore, Pakistan

**Keywords:** authentication, EEG signals of a deceased person, EEG signals, forged EEG signals, liveness detection

## Abstract

This work is aimed at investigating the potential risks linked to electroencephalography (EEG)-based person authentication and providing solutions to mitigate these issues. Authenticating a person by EEG involves verifying the legitimacy of the signals used for user identification. EEG signals serve as a biometric modality for authentication and verification. Additional biometric modalities, such as fingerprints or irises, are vulnerable to both fabrication and degradation over time, and illicit use of dead people's biometrics has sometimes been seen. EEG's intrinsic properties prohibit signal imitation or postmortem acquisition, making it more reliable than other biometric modalities. This research is aimed at investigating the most recent advancements in the domain of EEG signals, clarifying the current knowledge that impacts EEG-based authentication, and evaluating the emerging challenges. Many research publications have been collected to achieve this objective. By considering historical and recent efforts and achievements, this research also presents feasible resolutions to the emerging inquiries prompted by the ongoing advancements in EEG-based technology. The potential future application of EEG-based authentication has also been the subject of this scholarly discourse. A comprehensive collection of articles over the previous decade has been compiled to answer contemporary EEG signal research questions to get valuable insights. According to research findings, in February 2022, a significant milestone was achieved when the EEG signals of a deceased person were successfully captured for the first time in recorded history. However, this groundbreaking discovery may threaten EEG-based authentication. In addition, it is found that EEG-based authentication literature did not completely implement “liveness detection.” An updated approach for identifying liveness addresses novel challenges, that is, falsified EEG signals and a dead person's EEG signals for EEG-based authentication that have not been discussed in the literature. The suggested solutions put forward in this study have the potential to stimulate additional research in this area.

## 1. Introduction

Personal identification based on unique biological characteristics dates back to 1500 BC [1]. It is typically incorporated into authentication and identification. Authentication refers to the process of confirming a person's claim about their own identity, whereas identification involves the steps used to establish the precise identity of this individual. Physiological and behavioral biometrics are the two primary subfields of biometrics [2]. Biometric modalities that use physiological characteristics, including fingerprints and irises, are under the umbrella term “physiological biometrics.”

**Figure 1:**
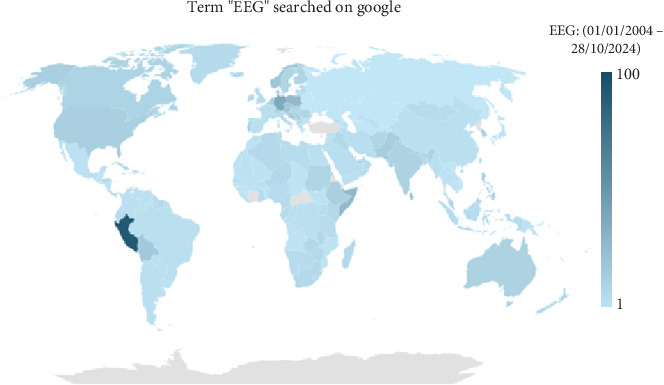
The blue shaded area shows that the word “EEG” was searched from all these areas in the last 20 years, Google.

In contrast, “behavioral biometrics” describes various types of biometrics based on how individuals carry out specific actions [2]. Electroencephalography (EEG) has seen a significant increase in its prevalence, mainly attributed to its extensive incorporation in scholarly investigations and real-world applications. The extensive use of EEG may be due to its noninvasive characteristics and straightforward data-gathering process. Its worldwide popularity is evident in Figure 1. The data in this image was obtained via Google Trends, where the search phrase “EEG” was entered, and the time range was limited to the last 5 years. The first use of EEG primarily centered on its application within the realm of neuroscience, namely, in identifying neurological disorders and investigating brain functioning. The broad use of EEG signals in biometric verification and identification is mostly attributed to the intrinsic distinctiveness of individual traits they possess [3].

**Figure 2:**
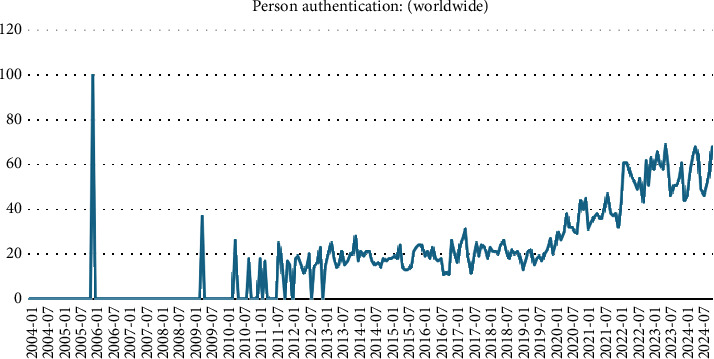
“Person Authentication” search results of the last 20 years, from Google.

**Figure 3:**
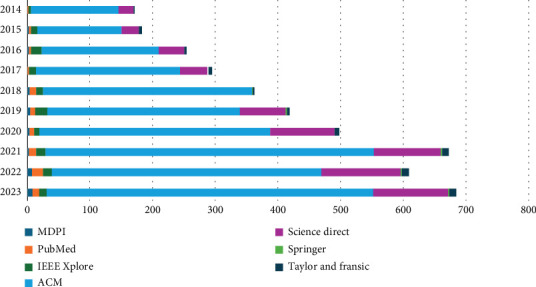
Articles published in the last 10 years on EEG-based authentication.

The reliability of EEG-based identification surpasses that of other biometric approaches, primarily owing to its capacity to effectively counteract counterfeiting attempts [4]. It has been ascertained that using EEG data from dead persons for authentication purposes is considered impractical owing to the lack of EEG signal emissions in deceased individuals. In contrast to the simple method in which the irises and fingerprints of a dead person may be acquired [5], EEG signals demonstrate higher efficacy than other biometric modalities. In recent years, there has been an apparent upsurge in using biometrics for person authentication.  Figure 2 depicts the upward trend in the prevalence of person authentication, as shown by the search trend data for “Person authentication” on the Google search engine.  Figure 3 shows a meteoric rise in scholarly works discussing “EEG-based authentication.” This finding indicates a burgeoning and heightened interest among scholars in this field. Over the last decade, several unsolved matters have emerged, necessitating a focused emphasis and subsequent resolution. EEG-based person authentication is used in many areas, such as two-step authentication [6], to enhance the security of mobile phones [7], particularly in brain-computer interfaces (BCIs) [8].  Table 1 provides the list of abbreviations and their explanation that are used in this study.

BCI provides an interface between the brain and the outer world [9]. Therefore, an individual can engage with the surrounding environment through brainwave activity without requiring bodily gestures or spoken instructions [10]. This technology is often seen in security, education, and medicine. Before executing commands received by brain signals, it is crucial to verify the origin of those directives [11]. EEG signals proved to be effective in implementing this authentication technique. In addition, distinct cryptographic keys are created by using the EEG signals of an individual [12]. As seen in Figure 3, there has been a recent and heightened interest among academic researchers in the “EEG-based authentication” field, which has resulted in a noticeable increase in the number of scholarly articles covering this topic. Significant issues in the last 10 years need careful consideration and effective solutions. One example pertains to fabricated EEG data generated via the generative adversarial network (GAN) technique. The illegitimate use of GANs by unauthorized persons has resulted in the production of counterfeit EEG signals. These fabricated signals have been exploited as genuine replacements, presenting a substantial risk to EEG-based authentication systems. Another potential concern is the EEG signals of deceased persons. The use of liveness detection has been suggested as a viable solution to the problem of distinguishing genuine from fraudulent EEG data [13]. The need for more research in this domain is justified due to its increasing prevalence, wide range of practical uses, and evolving complexities. To provide valuable insights to researchers and practitioners, we conducted an in-depth examination of past endeavors, present developments, obstacles, and prospective avenues for growth.

### 1.1. Significance

The use of EEG-based authentication is becoming increasingly prevalent in the domain of academic investigation, as depicted by data in Figure 3. The data is taken from well-known databases, that is, IEEE [14], PubMed [15], MDPI [16], Taylor & Francis [17], ScienceDirect [18], Springer [19], and ACM [20].

EEG signals are widely acknowledged for their exceptional robustness compared to other biometric modalities due to the prevailing belief that they cannot be generated postmortem [5, 21]. The use and study of EEG data have received considerable attention and recognition in both practical and scientific domains. Alternative biometric modalities' primary challenges are the proliferation of counterfeit photos generated by generative artificial intelligence (GAI) and the potential use of biometric data posthumously [22]. A suggested strategy for addressing the issue of counterfeit EEG signals is the implementation of a “liveness detection” method [23, 24].

The proposed liveness detection technique specifically addresses the issue of counterfeit EEG signals but does not address the problem generated using the signals generated by deceased individuals [13]. This distinction was made based on the prevailing belief at the time of the proposal of liveness detection for EEG-based authentication; it was believed that capturing EEG signals from deceased individuals was not attainable. In February 2022, a significant milestone was achieved when EEG signals from a dead individual were successfully recorded, marking a groundbreaking achievement in the chapters of human history [25] (discussed in Section 2). The significance of addressing this issue lies in the potential for enhancing liveness detection and considering the impact of EEG signals from deceased individuals to preserve the integrity of EEG-based authentication. This paper examines the challenges encountered by EEG-based authentication and anticipates the future potential areas of EEG-based authentication. Implementing robust procedures, such as liveness detection, is necessary for using EEG data for person identification to achieve a high degree of assurance.

According to ISO, 2023 liveness detection definition is: “measurement and analysis of anatomical characteristics or involuntary or voluntary reactions to determine whether a biometric sample is being captured from a living subject present at the point of capture” [26]. There are two primary goals of liveness detection: The first is to ensure that the person is alive at the point of capturing biometric information, and the second one is that biometric information must be pertained at that very time and should not be replayed or faked. Only one aspect of liveness detection is implemented in the literature in the case of EEG-based authentication, and the second point was never monitored or discussed as it was thought that EEG signals of a dead person could not be captured. There is a need to fully implement all the aspects of liveness detection that are presented by ISO standards.

### 1.2. Contribution

This study presents a systematic literature review (SLR), which includes a comprehensive survey of the current methods, trends, and technologies used for EEG-based authentication. The significant contributions of this review are summarized as follows.

• In this study, we evaluated EEG-based authentication techniques and emphasized the recent advancements in the domain of EEG signal processing.
• We examine how recent developments in EEG signal processing have affected EEG-based authentication. This is how fake EEG and deceased persons' EEG signals impacted EEG-based authentication.
• The issue of the deceased person's EEG signal for the person's authentication is addressed for the first time, adding novelty to this study. This is a crucial threat to liveness detection and has not been discussed in the literature earlier, as a dead person's EEG signals were captured for the first time in the history of the world in February 2022.
• To tackle these issues effectively, it is essential to employ the liveness detection approach documented by the ISO. Previous literature has not highlighted this topic.
• Given the nature of this review-based study, we have identified previously unrecognized gaps in knowledge that have emerged from our research, which are distinct and original contributions to the current literature. In this study, we have delineated innovative methodologies that may be used to uphold the resilience of EEG-based authentication.
• The primary aim of this study is to aid researchers in comprehending the significance of liveness in the context of EEG-based person authentication. Furthermore, the objective is to summarize the literature on EEG-based authentication during the last 10 years. Research articles are not limited to the previous 10 years; other years' papers have also been included to add pertinent information.

The following section provides a background study that includes information on brain signals, their frequencies, the function of these frequencies, different kinds of brainwaves, feature extraction approaches, and an overview of classification methods. The paper investigates potential vulnerabilities in EEG-based person authentication systems and presents solutions to mitigate these threats. The Methodology section provides a detailed account of how this research study was carried out. The Result and Discussion section presents the answers to the research questions, aiming at uncovering data insights. The Discussion section provides a concise overview of the results. The discussion part also includes an analysis of the future directions and limitations of the work. The Conclusion section serves as the last part of the work, summarizing the main points and offering further guidance for future scholars.

## 2. Background Study

Authentication is a procedural mechanism that entails the integrity of an entity. This pertains to a process by which an individual's authenticity may be validated. In each field where security is directly or indirectly involved, it is essential to prioritize its implementation [27]. The use of biometrics, passwords, and tokens may achieve the purpose. When compared to other approaches, biometric authentication exhibits a greater degree of effectiveness. EEG-based verification, made possible by recent technological advancements, has effectively reduced the potential risks associated with earlier methodologies. As an example, the perpetrator may use irises, which bear resemblance to fingerprints, after the death of a victim. However, the harmful use of EEG signals is impractical [24]. The use of brain signals in medical sciences and the investigation of psychological behavior may be traced back to 1875 [28]. Many techniques may be used to capture brainwaves. Various terminologies are attributed to brain signals based on the used methodologies, including intracranial electroencephalography (iEEG), magnetoencephalography (MEG), and stereoelectroencephalography (sEEG) [27].

The central nervous system (CNS), containing the brain and spinal cord, manifests a reaction to external stimuli while displaying two unique classifications of brainwaves. External stimuli, known as evoked or event-related potentials (ERPs), elicited one type of potential. The other type is created internally, without external stimuli, throughout both active and resting states [29]. According to the literature, EEG signals generated during the resting state exhibit reduced noise levels. The evoked-related potential (ERP) may be categorized into five distinct components: P100, N100, N250, P300 [30], and N400 [31]. Visual evoked potential (VEPs) are extensively used in academic research endeavors. Integrating visual and aural stimuli elicits distinct connectivity patterns within the brain. EEG data indicate biological processes, while physiological and behavioral cues enhance precision [32]. Additional multitask paradigms include activities related to visual perception, arithmetic processing, and graphic rotation.

**Figure 4:**
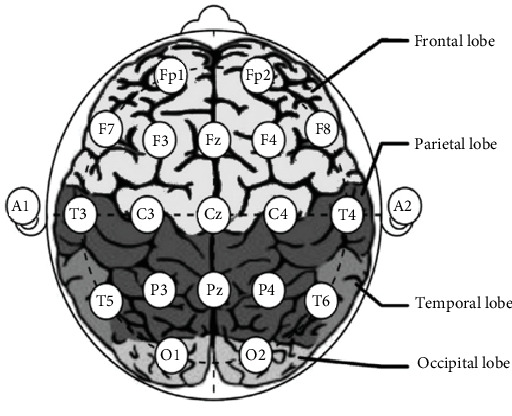
Placements of electrodes for capturing EEG signals. F electrodes capture EEG signals from the frontal lobe, C from the motor cortex region, T from the temporal lobe, P from the parietal lobe, and O from the occipital lobe [5].

The range of brain signal frequencies spans from 0.5 to 40 Hz. The frequencies are categorized into delta, theta, alpha, beta, and gamma bands according to their association with certain brain functions. Delta waves are generated during the phase of deep sleep, theta waves are generated during states of sleepiness, alpha waves are generated in a mentally relaxed state, beta waves are generated while the brain is actively engaged, and gamma waves are generated during the execution of various sensory processes [29]. Various regions of the brain perform multiple tasks. Hence, the frequency is integrated with the neural regions accountable for executing a particular cognitive function to comprehend a specific reaction [3, 5]. Various electrodes are placed directly on distinct regions of the scalp or brain to record brainwaves. The electrodes in question are often denoted as channels, labeled explicitly as FP1, FP2, F7, F3, Fz, F4, F8, T3, C3, CZ, C4, T4, T5, P3, Pz, P4, T6, O1, and O2, as seen in Figure 4. The F channels are used to record brainwave activity originating from the frontal lobe, a region of the brain associated with memory and emotional processing. P channels represent the parietal lobe, a cerebral area involved in cognitive functions such as problem-solving and attention. “T channels” often denote the brain's temporal lobe. This technology extends to face recognition, auditory perception, and memory retrieval. C channels are used to capture brain impulses originating from the sensorimotor cortex, a region of the brain responsible for executing mental processing and fine motor abilities [31].

Specialized headsets are necessary to capture brainwaves due to the relatively inconspicuous nature of EEG data compared to other biometric measurements. Various firms, including Neurosky, Google, and EMOTIV, provide headphones designed to capture brainwave data [33, 34]. Different recording techniques are used to collect EEG signals in both the resting state when individuals are not engaged in any specific task and the active state when individuals are undertaking a particular activity. Subsequently, the signals underwent preprocessing and classification. Support vector machine (SVM), *K* nearest neighbor (KNN), and deep learning (DL) methodologies have been commonly used in several research investigations for classification [35].

### 2.1. Role of Brainwave Frequency

During the examination of EEG signal frequencies, it became apparent that cosine similarity plays a crucial role in the differentiation of signals [36].

• The delta band had the least similarity when comparing various EEG readings.
• The delta frequency band exhibited distinctive characteristics and unique data that facilitated the identification process and was referred to as the steadier band.
• Some studies have shown that beta and gamma frequency bands perform better than others.
• A few other studies found that a higher level of complexity characterizes gamma waves and exhibits superior performance.

### 2.2. Biometric for Authentication

Authentication relies on pre-existing distinctive information about the individual. Scholars have examined three key aspects of biometric authentication: knowledge, ownership, and biometric traits [5]. Biometric variables provide a higher level of resilience than other factors, mainly owing to their intrinsic characteristics that render them impervious to theft or forgetfulness. The concept of universality pertains to the ability of a specific biometric component to be used for any individual. Permanence may be defined as the inherent attribute of stability and consistency over an extended duration. The biometric measurements of each individual displayed distinct traits, illustrating their uniqueness. Collectability refers to the ease with which a user may acquire or capture something [36]. To get EEG signals, specific steps need to be implemented. For instance, the individual must maintain a state of calmness and engage in predetermined cognitive activities that only involve brain activity while refraining from any movement of other bodily parts, for example, engaging in mental imagery activities, word association exercises, and internalized singing exercises. The EEG signals of a participant were captured with a specialized headset, subjected to preprocessing techniques, and then classified.

Authentication refers to the procedural steps involved in confirming an individual's identity and evaluating the accuracy of their self-claimed identity. Authentication is often performed using a binary classifier [37]. The model must be equipped with EEG data obtained from a person to facilitate training. The first stage of training is often known as the registration phase. The individual's brainwave patterns were recorded and compared to existing EEG data during the experimental phase. There was a prevailing belief that EEG signals were only obtainable during an individual's vitality, rendering them impervious to unauthorized access or theft. The uniqueness of these signals in terms of cerebration makes them more robust compared to other biometric approaches [27]. The use of EEG-based authentication to bolster system security has been documented in many sectors of computer science. In a particular research, EEG was used as an integral component of a two-tier authentication approach inside a mobile device application [6]. The domain of EEG-based authentication is gaining prominence in research, as seen by the growing number of studies conducted in this area, as shown in Figure 3 and Table 2.

BCIs have gained significant attention due to their potential to construct a direct neural interface between the human brain and machines or computers, eliminating the need for external interventions [29]. EEG waves are extensively used in incorporating BCI for data processing and person authentication. Some researchers have used EEG signals to generate cryptographic keys, while others have used them to enhance mobile app security measures.  Table 3 summarizes the research areas that have used EEG-based authentication in different activities [46, 47].

Table 3 describes research on EEG-based biometric authentication and identification that used various datasets, EEG channel configurations, sampling frequencies, and processing methodologies to enhance biometric security applications. Kjeldgaard and Dyhr [38] made mobile EEG–based biometric authentication. A RESTful web interface for mobile biometric authentication was built using power spectral density (PSD) and wavelet analysis on short EEG recordings from the P7, P8, O1, and O2 channels within a self-constructed dataset.

EEG signals were shown to be beneficial for mobile biometrics, a novel approach at that time. Lin et al. used a self-developed dataset to study EEG-based BCIs using ERP and discriminated and stylized diffusion (DS) fusion. Using FP1, FP2, Pz, and Oz channels at 250 Hz, they used NBC and SVM ensembles and obtained 75% classification accuracy, up from 60% with DS fusion. This research shows how EEG may improve BCI categorization for interaction [39]. The frequency used for EEG-based verification varied from 150 to 256 Hz, as shown in Table 3. The authors attained an accuracy rate of 91.1% in EEG-based authentication by using the random forest (RF) technique [45] and 95% accuracy by using mean curve length (MCL) [42]. The visual geometry group (VGG) achieved accuracy rates of 88%, 87%, and 90% for 8, 16, and 64 channels, respectively [40]. The principal component analysis–support vector machine (PCA-SVM) decoder achieved an accuracy of 95% on the same dataset. Yang, Libert, and Van Hulle used bagging, a machine learning (ML) technique, in conjunction with long short-term memory (LSTM) to analyze eight-channel EEG data. Their approach yielded an accuracy of 92.5% [41]. Tian et al. attained an accuracy of 98.5% by using a graph convolutional neural network (GCNN) on the publicly accessible dataset “PhysioNet” [43]. Research has shown that EEG is not influenced by auditory stimuli, indicating that it is genre independent. Researchers do not need to be concerned about aural cues while conducting EEG-based authentication [44]. Cooper et al. attained a perfect accuracy rate of 100% in EEG-based authentication by using independent component analysis (ICA) and neural network (NN) [37]. Kjeldgaard and Dyhr showed that EEG can be used with mobiles for biometric authentication [38]. EEG signals are also used in BCI applications and cryptography [8, 12, 39].

### 2.3. Datasets

The following datasets are primarily used for EEG-based authentication in literature.

• The PhysioNet EEG motor imagery dataset was used in most studies with 64 channels and a sampling rate of 160 Hz. Their diversity in tasks has attracted considerable research attention [48].
• The BCI Competition III Dataset has been widely used for this purpose. It has 62 channels and a 250-Hz sampling rate [49].
• BCI Competition IV dataset, 22 EEG channels, three EOG channels, and a sampling rate of 250 Hz.
• University of California, Irvine Knowledge Discovery Database (UCI KDD) EEG-based dataset, 64 channels, and 256-Hz sampling rate [50].
• Australian EEG database, 11-year study: sampling rate 167 Hz.
• DEAP: 32 channels, 512-Hz sampling rate (emotional states were recorded in this dataset) [51].

EEG waves are primarily captured in resting states when a person is relaxed while sitting on a chair, and both eyes are closed. Visual stimuli are also used to capture EEG signals. Rapid serial visual presentation (RSVP) is a protocol used for recognition, where a person is shown various pictures. After finding a known object or picture, the brain emits a P300 ERP. The most widely used protocol for EEG-based authentication is the steady-state evoked potential (SSVEP), a visual stimulus in which a periodic visual stimulus is introduced at 4–60 Hz. Acoustic stimuli, mental tasks, and multiple tasks are also used when recording EEG signals [5].

Table 4 shows the data collection methods used in the literature and their strengths and weaknesses.

There have been many other approaches to data collection that have been reported in the literature, with the EMOTIV EPOC headsets being one of the more famous ones [38]. It is simple to use and very cost-effective and may be simply used in situations that occur in the real world. On the other hand, its signal quality is poor, and it is prone to artifacts and increased noise levels in EEG data. When trying to improve the quality of the signal, it is advised that dry electrodes be used [39, 41]. For this purpose, EPOC dry-contact wireless headphones are used. Even through hair, they are able to determine and maintain constant connections. On the other hand, they were still unable to provide signal quality that was similar to that of gel-based systems. Due to the insufficient signal quality, the system's accuracy is reduced when it is used in a dynamic environment. A system with 64 channels gives improved accuracy in the identification of brain patterns [40], in comparison to dry electrodes. These devices have electrodes with a high density, which allow them to deliver a substantial amount of spatial data. It is not possible to use this configuration for mobile apps since the procedure of setting it up is quite long. The practicality of these systems in applications that take place in the real world is improved by reducing the number of channels that they include.

Other datasets like University of California (UCI) [12] and PhysioNet motor movement/imagery [37, 43] provide task-oriented data with larger sample sizes. The UCI dataset emphasizes motor imagery and visual stimuli and is statistically stable, whereas the PhysioNet dataset offers high temporal accuracy and a large sample size. Because both datasets were gathered under controlled conditions, their use in noisy and dynamic circumstances is limited. Simulation of environmental noise may help enhance datasets and make them more realistic. Gel-based disk electrodes function well because of their good signal quality, reliable data, and optimal scalp contact [42]. However, gel-based systems need lot of time. In order to mitigate this problem, an innovative wearable design should be adopted to balance between signal quality and mobility. It can be inferred from the results that all the methods have some merits and demerits. Advancements in hardware technology may enhance the data collection process so that computational cost and ease of use are not compromised.

### 2.4. Preprocessing

The following paragraphs present a general description of the various kinds of preprocessing done for EEG data to authenticate a person. In the time domain, the data were in the waveform. Statistical methods and signal-smoothing techniques were used in the time domain. Mean, median, variance, and normalization are well-known time-domain analysis techniques [27]. In the frequency-domain analysis, EEG signals were converted into the frequency domain using a fast Fourier transform (FFT). The frequency-domain analysis mainly uses filters and frequency-spectrum estimation. Widely used filters are the Chebyshev (low pass) and Butterworth filters [52]. In the time-frequency-domain analysis, the time-domain EEG signals were combined with the frequency-domain information of the EEG signals. It transforms the signal from a one-dimensional form into a two-dimensional shape. It can express the features from both domains. Some examples [53] of time-frequency-domain analysis are short-time Fourier transform (STFT), wavelet transforms, and wavelet packet decomposition (WPD). In recent EEG-based authentication studies, researchers have captured signals from multiple electrodes. The spatial domain is more robust than other methods for removing noise and artifacts. Spatial domain analysis can be done by using [27] principal component analysis (PCA), ICA, common average reference (CAR), and Laplacian filter. Nonlinear dynamics determines the activity state of the brain using nonlinear dynamic principles. The methods proposed in the literature include phase synchronization and brain connection networks [54]. Researchers used spatial, time-, and frequency-domain filtering in preprocessing [37, 38, 44].  Table 5 shows the preprocessing steps used in various studies. The following table shows which preprocessing methods have been adopted in the literature along with their strengths and weaknesses.

Butterworth filtering was used in many researches to separate frequency ranges like 0.5–40 Hz or 1–30 Hz to remove noise-causing high-frequency components [38]. Butterworth filters concentrate on lower frequency bands where cognitive and motor information is highlighted and has smooth frequency response and little signal distortion. Unfortunately, these filters may remove potentially helpful high-frequency components. When dealing with real-world applications that include fluctuating signal ranges, adaptive filtering techniques such as least mean square (LMS) are recommended. Another alternative is filtering based on wavelets, which minimizes distortion and maintains signal structure, especially close to the signal's edges. A research used canonical correlation analysis (CCA) [39] to identify and reduce artifacts and improve signal quality [39]. CCA reduces noise but is computationally demanding, unlike ICA. To reduce computing effort and improve noise reduction, ICA and artifact categorization might be combined.

EEG data is segmented into shorter temporal frames using sliding window segmentation [43] to capture patterns over time and enhance data volume. This approach creates numerous trials every session, making it effective for training models with little data. However, overlapping sliding windows, especially those with 75% overlap, may add redundancy and complexity. A smaller overlap percentage or batch processing may minimize computing costs while keeping segmentation advantages. Fixed segmentation captures temporal information but risks losing long-term temporal information. To preserve short- and long-term patterns, adaptive segmentation with varying window lengths is advised.

### 2.5. Feature Extraction

Signals in the temporal, spatial, and spectral domains may all be used to infer features [27]. For the time-domain signals, normally, the autoregressive (AR) method is used for feature extraction; for frequency-domain PSD, for time and frequency-domain wavelet coefficients, and for spectral frequency domain, common spatial coherence is employed by the researchers mostly.

**Figure 5:**
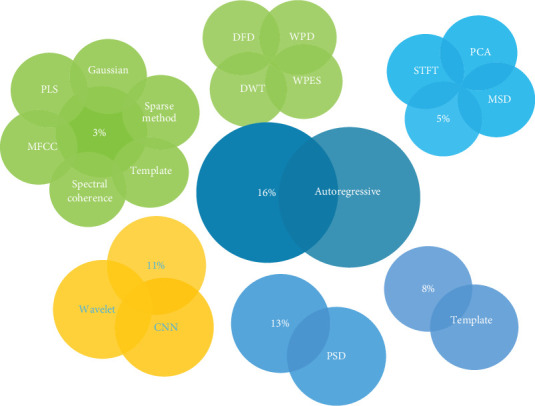
Feature extraction techniques used in literature for EEG-based authentication.

The feature extraction process may be accomplished using the FFT. An AR parameter model used the FFT algorithm to derive a singular spectrum. The foundation of this concept is rooted in the principles of a linear regression model [55]. PSD is an additional approach for extracting features, which characterizes signal intensity distribution in a time series depending on the frequency [56]. The common spatial pattern (CSP) technique is well recognized as a prominent approach for extracting features from EEG data. This approach is often used when there is a need for binary classification [29]. Subsequently, the geographic attributes of each category were ascertained. Motor imagery (MI) EEG signals perform superior to other feature extraction techniques, with a classification accuracy of up to 95% [57]. Nevertheless, this approach is unsuitable for multiclass classifications. Phase synchronization is a technique for extracting features from EEG data, explicitly examining the interplay among different channels [58]. In most studies, the researchers employ PSD and AR for EEG-based authentication, as shown in Figure 5. According to [59], the primary purpose of using PSD is to ensure the stability of features. Nevertheless, it is not conducive to analyzing unstable signals, particularly those in the time domain. The Fourier transform is adequate for analyzing stationary and narrow-band signals but unsuitable for nonstationary signals. It significantly contributes to the exacerbation of noise sensitivity [59].  Table 6 provides information about feature extraction methods used in the literature, their strengths, weaknesses, and methods to mitigate the weaknesses.

### 2.6. Authentication Methods

Following are the authentication methods used in the literature.

#### 2.6.1. Classification Method

**Figure 6:**
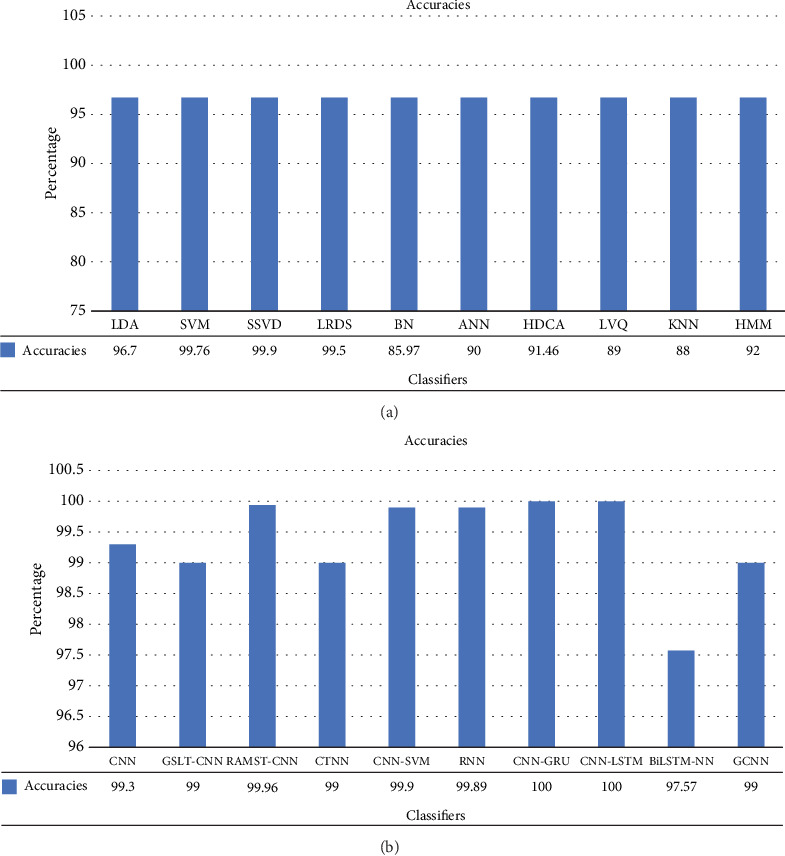
(a) Machine learning algorithms' accuracies. (b) Deep learning algorithms' accuracies.

Similarity-based approaches have been used in several research for authentication, with the authors comparing the two vectors. Individuals were admitted if their similarity score was above the predetermined threshold value; conversely, they were denied if their score fell below the threshold. The Euclidean distance, crosscorrelation, cosine distance, Manhattan distance, and dynamic time wrapping were used to compare directly [60]. Other methods used for EEG-based authentication include SVM [40, 61] linear discriminant analysis (LDA) [62], hidden Markov model (HMM) [63], artificial neural network (ANN) [53, 64], KNN [65], support vector data description (SVDD) [66], RF [67], Gaussian mixture (GM) [68], low-rank sparse decomposition (LRSD) [69], and SD network (BN) [70]. Linear vector quantization (LVQ) demonstrated an accuracy of 89% [71], while hierarchical discriminant component analysis (HDCA) achieved 91.46% [72], as shown in Figure 6(a).

Artificial intelligence has seen a notable transition, as shown by the data in Table 7. This transformation may be attributed to the progress made in DL methods, which have substantially improved the dependability and accuracy of several domains within AI. The process can extract characteristics from unprocessed data. Deep EEG methods used for EEG-based authentication include convolutional neural network (CNN) [72] and recurrent neural network (RNN) [35, 80]. LSTM and gated recurrent units (GRUs) have been used extensively. Residual and multiscale spatiotemporal convolutional neural network (RAMST-CNN) [81] gained an accuracy of 99.96%. The combination of CNN and LSTM [77] provides more precision than the other models, and bidirectional long short-term memory with neural network (BLSTM-NN) [78] has also shown good performance with an accuracy of 97.57%. Similarly, CNN-GRU demonstrated good performance (98% and 99.17%, respectively). CNN-SVM [76] provided an accuracy of 99.1%. Global spatial and local temporal convolutional neural network (GSLT-CNN) [75] achieved 99% accuracy, as seen in Figure 6(b). The convolutional tensor train neural network (CTTNN) demonstrated excellent performance, even when trained with limited data. This stands in contrast to other DL models [82]. GCNN outperformed all other DL models by showing an accuracy of 99.98% [79].

#### 2.6.2. EEG-Based Biometric Classification

The literature reveals that features were retrieved throughout the EEG-based authentication process to differentiate each person within the group. Everyone in the group was regarded as an independent entity.

Supervised learning for user authentication was employed along with the classification of individuals based on their identities [83].  Table 3 presents a comprehensive summary of the research that used the EEG-based authentication approach.

#### 2.6.3. EEG-Based Biometric Cryptosystem Authentication

The characteristics of the EEG signal for each person are used to distinguish the person as a separate class during the authentication process. The problem arises when artificial EEG signals are produced using a GAN based on these unique characteristics or features [84]. Biometric cryptosystems have addressed fake EEG signals and security problems [65]. The “key” is the vertex around which cryptography revolves around. Biometric cryptography can be divided into three steps: key-combining biometric cryptosystems, [46], key-generating biometric cryptosystems [47, 85, 86], and key-binding biometric cryptosystems [87, 88] as listed in Table 8; data from the research articles were analyzed under similar paradigms to find answers to some research questions.

Task-independent person authentication is used for authentication, in which any task can be performed for authentication purposes; only the person should be in a calm state of mind [69].

### 2.7. Threats to EEG-Based Authentication

EEG signals have been regarded as more resilient than other biometric modalities due to their inherent resistance to forgery or theft. Nevertheless, after recent developments in research, significant challenges exist to the viability of EEG-based verification. The following paragraphs address some of the crucial problems.

#### 2.7.1. Fake EEG Signals

The primary application of the GAN included data augmentation, namely, the generation of EEG signals from limited data sources. This data is used in several applications, such as motor imager applications, emotion identification, epilepsy studies, and BCI applications [89]. In a particular research investigation, the authors provided evidence of the effectiveness of using synthetic EEG signals to circumvent security measures linked to EEG-based person authentication. Subsequently, the authors introduced a technique to identify liveness to counteract counterfeit EEG data for person identification. Their approach seeks to maintain the robustness of the EEG signals [13].

#### 2.7.2. Deceased Person's EEG Signal: In Most of the Research Studies, It Is Stated

• “These signals can only be recorded if the living individual is functional and receptive. It means that there is no possibility that a non-living brain can produce EEG signals, which in hindsight prevents attackers from stealing this characteristic from the user” [90].
• “… EEG has emerged as a good candidate for individual identification because of its advantages such as universality, intrinsic liveness detection capability, and robustness against attacks” [91].

This conviction is undermined when EEG signals from a deceased individual are recorded, exhibiting similarities to those seen during the individual's living state. A deceased person's EEG signals are a new threat to EEG-based person authentication. In a research study, for the first time in the history of the world, EEG signals of a human are recorded after his clinical death [25]. A study done in 2013 examined the neurophysiological condition of the brain in the immediate aftermath of cardiac arrest. The study included using rats as subjects, focusing on continuously recording EEG data. The study investigated power density, coherence, directed connection, and crossfrequency coupling in the brains of rats after experimentally causing cardiac arrest. The data were acquired with a continuous EEG methodology. A considerable increase in synchronized gamma oscillations is seen during the first 30-s period after cardiac arrest, before the onset of an isoelectric EEG. The gamma oscillations seen after cardiac arrest had a wide frequency range and demonstrated synchronous activity. Additionally, they showed heightened anterior–posterior connectivity and phase synchronization, including theta and alpha oscillations [92]. EEG signals were observed after the animals' demise, exhibiting resemblances to the EEG patterns recorded during the animals' state of living.

**Figure 7:**
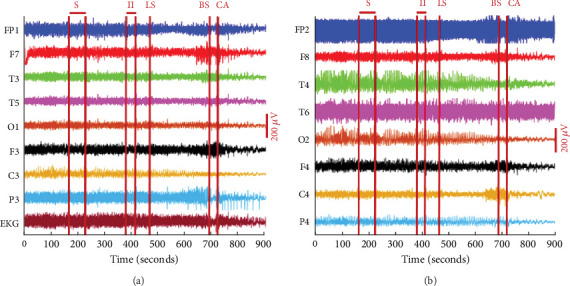
EEG signals of the person before and after death. (a) EEG signals from the left hemisphere and (b) EEG signals from the right hemisphere. Sixteen electrodes were placed on the person's scalp at positions FP1, FP2, F7, F8, T5, T6, O1, O2, F3, F4, C3, C4, P3, and P4. The signals extracted from these locations are referred to as channels. On the top of the figure, S shows seizure, LS shows suppression of left cerebral hemisphere activity, BS shows suppression of bilateral cerebral hemisphere activity, and CA shows cardiac arrest [25].

In February 2022, a revolutionary experiment was undertaken, representing the first occurrence in documented history, whereby an individual of 87 years of age, afflicted with cardiac arrest after a catastrophic subdural hematoma, was submitted to said experiment. The main distinction between the prior investigation and the human research is the implementation of cardiac arrest. In the first experiment, cardiac arrest was induced in rats, whereas in the second experiment, an individual was taken to the hospital after a fall and subsequently underwent cardiac arrest [25].  Figure 7 illustrates that the signals seen after cardiac arrest exhibit similarities to those observed before cardiac arrest across many channels. When a person dies, unlike with electrocardiography (ECG), there is no discernible drop in EEG signals.  Figure 7 shows the continuity between the EEG signals recorded throughout a person's life and those recorded after death. The issue at hand is important, as it pertains to the potential exploitation of postmortem EEG signals for person authentication.

#### 2.7.3. Replay Attacks

This entails capturing the EEG signals of an authorized individual and then using them to bypass authentication. It may provide a significant risk to EEG-based authentication systems. Recording EEG signals without an individual's consent is challenging since it necessitates the placement of a headset on the subject's head in a certain manner. However, if an individual is asleep or unconscious, their EEG signals may be captured.

#### 2.7.4. Spoofing

Despite the uniqueness of EEG signals, a proficient individual may replicate them using GAI. The generation of synthetic EEG signals has emerged as an expanding field. It has uses in both personal authentication and medicinal fields. An adept individual may use the EEG signals of an authorized person and employ GANs and variational autoencoders (VAEs) to analyze the distribution and patterns, subsequently generating EEG signals. Advanced generative models can collect temporal and spatial data and generate high-quality signals.

### 2.8. Mitigation Strategies

The literature presents many mitigation solutions for addressing problems in biometric domains.

#### 2.8.1. Liveness Detection

Liveness detection means knowing the person whose biometrics are being used is alive. Ensure that his fake or illegally acquired biometrics are not used, that is, stolen pictures, faked, or using biometrics after a person's death. To authenticate a person using his biometric traits, his biometric must be verified and tested before authenticating him as a verified user. The ISO and the International Electrotechnical Commission (IEC) defined liveness and liveness detection [26].

• Liveness: “quality or state of being alive, made evident by anatomical characteristics, involuntary reactions, physiological functions, voluntary reactions, subject behaviors, or any combination of these [26].”
• Liveness detection: “measurement and analysis of anatomical characteristics or involuntary or voluntary reactions to determine whether a biometric sample is being captured from a living subject present at the point of capture [26]”.
• Presentation attack: “presentation to the biometric data capture subsystem to interfere with the operation of the biometric system [26].” Reproducing speech or brain/heart signals, rubbery fingers with genuine user fingerprints, showing user photographs (printed or on-screen) to a facial recognition system, etc. are threatening inputs that imitate authentic human features. The primary objectives of liveness detection include two aspects. The first objective pertains to verifying the vitality of an individual, ensuring that they are alive. The second objective involves ensuring the timeliness of the input, explicitly confirming that the biometric attribute being collected is obtained in real time and not a result of replay or forgery [13].

#### 2.8.2. Liveness Detection for EEG Signals

Until 2018, no work has been done for the liveness detection of brain signals. “However, there are no works on liveness detection for brain signals since it is commonly assumed that these signals possess an intrinsic liveness property” [13, 93]. The issue of detecting the liveness of brain signals was first brought to attention by Javad et al. in the year 2021. Their only emphasis was on artificial EEG signals. At that juncture, the EEG signals of deceased individuals were not recorded. They did not fully implement all the aspects of the liveness detection definition presented by ISO. In this research study, we have identified a research gap so liveness detection can be fully implemented, and all its aspects must be carefully monitored.

#### 2.8.3. Multifactor and Multimodal Authentication

Person authentication should not rely only on EEG signals or a single biometric modality. They should also consider the irreplicable measurements with biometrics, such as body sweat monitoring systems, pulse rate and patterns, face expressions, and vocal characteristics. Synthetic EEG will not bypass the authentication procedure.

#### 2.8.4. Signal Consistency and Anomaly Detection

Generative models are unable to attain consistency about microfluctuations in brainwave signals. Such methods may assist in identifying synthetic EEG signals. Authentic EEG data exhibit spontaneous variations resulting from a confluence of intrinsic brain mechanisms, including continuous cognitive functions, autonomic control, and other neurophysiological responses. These microdynamics are unique to each person and may fluctuate considerably even over brief intervals, complicating the ability of generative models to duplicate them with precision continuously.

## 3. Methodology

A systematic review was done to uncover pertinent studies focused on EEG-based authentication. This section will provide an overview of the study's methodology and findings. To ensure a thorough examination of existing literature, this study adopts a systematic methodology that encompasses the identification, collection, classification, and review of contemporary academic articles relevant to the designated research topic. We have used a systematic strategy by sticking to the defined inclusion criteria for the selected field to validate the literature review results.

This technique is aimed at clarifying the methods used in identifying important aspects of the current investigation. The primary aim of this study is to do a thorough examination of the existing body of literature about EEG-based verification using the following procedural steps:

Step 1. The process of developing research questions is essential. The process entails narrowing down a wide field of study into something that can be studied comprehensively. This process requires careful consideration of the variables, concepts, and scope.
Step 2. The examination of the data and identification of important articles.
Step 3. Establishment of conditions for inclusion/exclusion of research articles.
Step 4. The procedure of collecting data or conducting observations to get information for academic study.
Step 5. Analyzing and interpreting data to draw conclusions and draw inferences.

### 3.1. Formulating Questions

The primary objective of this study is to examine the existing literature on EEG-based authentication. This research is aimed at analyzing all of the techniques and challenges of the subject over the specified period. This study is aimed at thoroughly investigating authentication techniques using EEG data and tackling the unresolved problems within this field. To achieve this objective, several research queries are investigated to address these problems efficiently. The following questions have been formulated for this research investigation.

RQ1. What are the application areas of EEG-based authentication?
RQ2. Which algorithms yield outstanding EEG-based authentication results? Which authentication algorithm do researchers most frequently employ?
RQ3. Which feature extraction techniques are mostly used for EEG-based authentication?
RQ4. How have the challenges in EEG-based authentication been addressed in the past? Which approaches were used?
RQ5. What is the credibility of EEG-based authentication based on recent research on EEG signals? Which open challenges must be addressed?
RQ6. What are the future areas for EEG-based authentication?

### 3.2. Data Examination Based on Keywords

**Figure 8:**
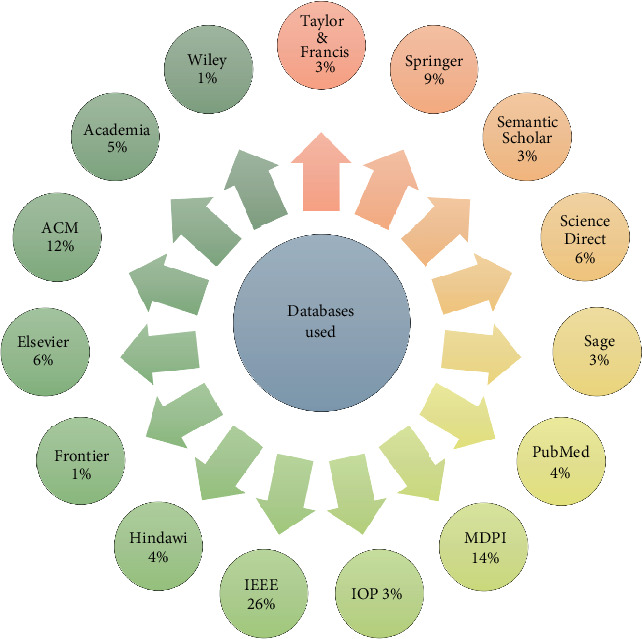
Databases used in this research study for downloading research papers.

The search technique was designed to consist of two distinct stages: the initial stage and the secondary stage. During the first search stage, candidate papers were selected by a combination of our own expertise and computerized database searches using a predetermined search phrase. The researchers searched for relevant studies and publications in EEG-based authentication using well-known databases, as seen in Figure 8. The second phase of the search process included reviewing the references cited in the primary publications identified during the first stage to identify more potential studies. This methodology has been iterated until no more relevant research has been identified. The methodology used for generating the search terms was as follows:

• Extract significant phrases from the questions.
• Identify several synonymous and alternative spellings for important phrases.
• Using the Boolean OR operator allows for using pertinent spellings and synonyms.

The Boolean operator AND should be used to establish a connection between the primary keywords. A comprehensive search for relevant research publications was conducted across many platforms to fulfill the study goals and address the research questions. The downloaded articles only consisted of publications in the English language. We have used these terms and expressions in our search for scholarly articles: “EEG-Based Authentication” OR “Person's Authentication,” “EEG as Biometric” OR “EEG Signal,” AND “EEG-based Authentication” OR “Brain Signals” OR “EEG Signals.” Many databases have been investigated as potential sources for scholarly papers, including Hindawi, Scopus, Web of Science, IEEE Xplore Digital Library, Semantic Scholar, PubMed, MDPI, ScienceDirect, and SpringerLink. A total of 150 research publications were retrieved, of which 100 were chosen based on the specified criteria.

**Figure 9:**
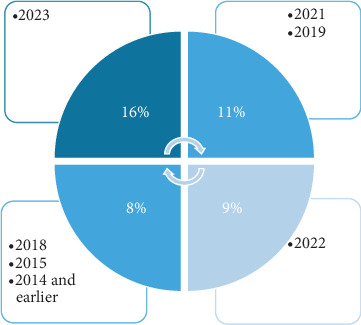
Percentage of the articles used in this study, from the last 10 years.

**Figure 10:**
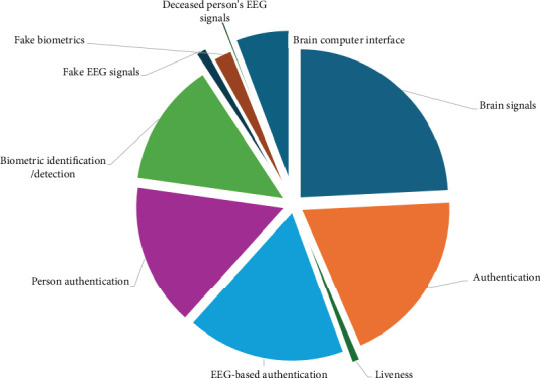
Selected article's percentage from different categories.

The selection process included the consideration of a certain journal or conference and the application of predetermined inclusion and exclusion criteria to relevant research studies. This study is aimed at selecting scholarly papers published over the last decade comprehensively. Emphasis was placed on recent publications, as seen in Figure 9, which presents the distribution of articles used from each year within the previous 10 years. This research also uses pre-2014 articles to deliver important information. While downloading the articles, the keywords most essential for us were “Brain signals” and “EEG-based authentication.” Several articles against the keywords used can be shown in Figure 10. A limited number of studies are available on liveness detection and EEG data analysis from deceased individuals. The limited research in this field has resulted in a scarcity of studies on EEG signals of dead individuals, with just one research paper now accessible. There is a research gap in using EEG data from deceased individuals for EEG-based authentication. Furthermore, implementing a liveness-detecting technique is necessary to address this emerging concern.

### 3.3. Inclusion and Exclusion Criteria

**Figure 11:**
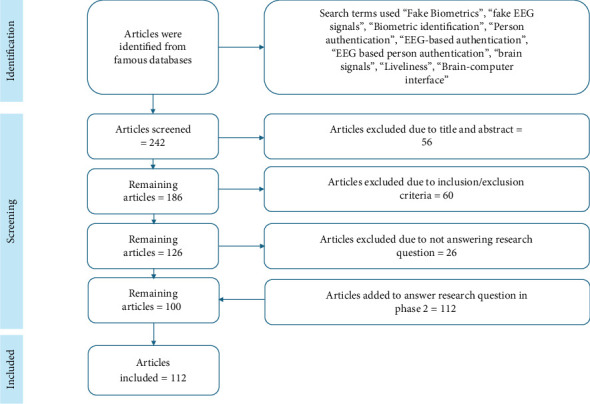
Process of article selection.

The primary materials were first selected based on assessing the study title, keywords, and abstract. During this step, only those articles that were completely irrelevant to the study field were rejected and excluded. Full articles have been gathered and screened following the criteria for inclusion or exclusion in the first selection process. In cases where a question arises about the inclusion or exclusion of a particular article, the article is forwarded to another reviewer for further evaluation. In summary, as previously indicated, one author conducted the main search inside the designated electronic libraries assigned to him. The other author screened the selected articles. Each article was screened considering the title, pertinent keywords, and abstract. After the first analysis, an in-depth review was conducted of the publications according to the criteria for inclusion and exclusion, as seen in Figure 11. The suggested taxonomy is characterized by its explicit requirements and rigorous evaluation methodology. In the context of researching and analyzing research papers, the criteria for selecting articles are summarized as follows:

• Academic publications throughout the time frame spanning from 2014 to 2023.
• Research papers on EEG-based authentication methodologies and procedures.
• The selection of suitable publications in the EEG field is facilitated by considering specific criteria, such as technical excellence.
• Articles published before 2014 can also be included if they provide significant information that necessitates their inclusion.

### 3.4. Data Collection

The necessary data was obtained from the chosen research sources to address the research questions comprehensively. This might be seen as the preprocessing step for a review article. Because at this stage, there is still no action taken, and just information is gathered. Two authors have examined every preliminary manuscript included in the review. One reviewer thoroughly examined the papers to extract the necessary data, while another scrutinized them as a verifier. The primary duty for obtaining the required data rested with the main data extractor, while another reader's role was to assess the suitability of the acquired data. We tried to get the listed data from each research article.

• The study's source, whether it be a journal or conference, should be included along with a complete citation.
• The categorization of the research type.
• The main subject of the research article.
• A comprehensive overview and summary of the article, focusing on the methodology used and the databases utilized.
• Quality of article
• The article aligned with the research questions.

### 3.5. Study Selection Based on Selection Criteria

**Figure 12:**
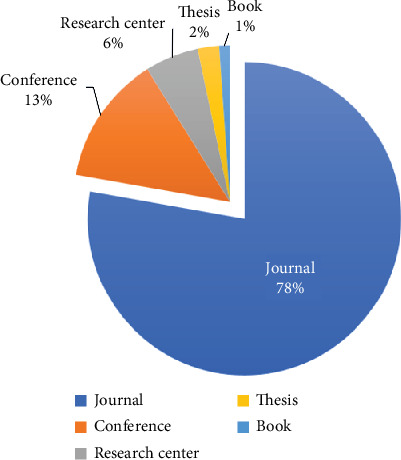
Selected article's percentage from different categories.

First, 222 research papers were downloaded using the keywords mentioned earlier, and 100 were selected for research. Among the 100 selected articles, 78% were journal papers, as shown in Figure 12. The remaining articles were conference papers and information from research centers, such as information about various datasets used in literature and some thesis research containing necessary information. Study selection for a review article is comparable to feature extraction, which involves selecting the most relevant publications to gather data insights to address the research objectives. The following steps are considered for selecting research articles.

• We have eliminated duplicate articles.
• Found the quality of the research papers.
• Papers that are relevant to the authentication of a person are included.
• Papers that are relevant to EEG signal processing are included.

Papers that have important information regarding novel EEG research are included.

• Excluded papers that show no relevance to the research objective, directly or indirectly.
• Papers that have no precise results or outcomes are excluded.
• Excluded papers in which the area of research is not clearly defined.
• Excluded the papers in which results are not explicitly discussed.
• The papers were thoroughly reviewed, and the results are summarized in Tables 3 and 9.

## 4. Result and Discussion

This section discusses the research questions and their answers by exploring the literature and devising new insights into data.

### 4.1. Application Areas of EEG-Based Authentication

This subsection helps us in finding the answer to the research question “RQ1: What are the application areas of EEG-based authentication?”

#### 4.1.1. Application Areas in Literature

As listed in Table 3, EEG-based authentication is primarily used in mobile technologies [33] and BCI systems [35]. Other areas include the introduction of isolation forests and local outlier factor classifiers, fast EEG–based biometrics for person verification, and real-time data implementation. It has also been used to conduct experiments in new dimensions, such as the effect of auditory stimuli and the Internet of Things (IoT) [8]. EEG-based identification can be done regardless of the task that is being performed with an accuracy of 99.3% [69]. Multitask EEG-based authentication is found robust against the instability of signals with an accuracy of 94.2% [72]. Applications domains of EEG-based authentications are summarized in Table 9.

#### 4.1.2. Potential Application Areas in the Future

There can be many potential applications of EEG-based authentication systems as they provide a unique form of a person's identification.

##### 4.1.2.1. BCI

BCI provides a bridge between the brain and external devices. EEG-based authentication can enhance security in these devices, by ensuring only the legitimate user can have access to the devices.

• EEG-based authentication can restrict illegitimate users from accessing sensitive devices, that is, medical devices, and assistive technologies.
• With the help of EEG-based authentication, continuous monitoring can be done throughout the session.
• EEG-based authentication can be used with BCI for cognitive state monitoring to check the user's mental state and generate an alarm in case of any dangerous situation.

##### 4.1.2.2. IoT-Based Authentication System

EEG-based authentication can be used in a wide range of IoT devices, as they provide a unique way to user's identification.

• EEG-based person authentication may regulate access to IoT-enabled home equipment for secure smart home management.
• Remote monitoring gadgets may also be used in conjunction with home security systems. EEG-based authentication will guarantee that only the authorized user may access monitoring equipment.
• EEG-based authentication enables safeguard access to machinery in the industrial sector and critical data systems.
• The use of EEG-based authentication has the potential to enhance security and robustness across several domains, hence contributing to an improved user experience. Future researchers may use these application domains.

### 4.2. Best Algorithms for EEG-Based Authentication

This subsection helps us find the answer to the research question RQ2: Which algorithms yield outstanding EEG-based authentication results? Both the shallow classification and DL methods demonstrated outstanding performance. In some cases, DL algorithms outcast ML algorithms, as shown in Figures 6(a) and 6(b). From these diagrams, it is evident that the accuracy of the ML algorithms ranged between 75% and 100%. The accuracy of the DL algorithms ranged from 96 to 100%. This shows that the overall performance of the DL algorithms was much higher than that of the shallow approach. Commonly used DL algorithms are CNN [72], RAMST-CNN [81], GSLT-CNN [75], CNN-SVM [75], RNN [35], CNN-GRU, CNN-LSTM, BLSTM-NN [78], and GCNN. All these algorithms achieved accuracies between 97% and 100%. However, in some cases, ML algorithms, such as SVM [76], SVDD, and HDCA [72] have shown remarkable results. Commonly used DL algorithms are the VGG and the CNN [72].  Table 10 provides the performance comparison of various algorithms on the same dataset, that is, PhysioNet for EEG-based person authentication.

Accuracies of classification algorithms vary due to the feature extraction techniques. By using PSD and spectral coherence connectivity (COH), Mahalanobis distance–based classifier achieved remarkable accuracies, that is, 100% and 98.83%, respectively. Mahalanobis distance–based classifier is a statistical-based classifier. DL algorithms also showed remarkable performance; that is, CNN-LSTM achieved an accuracy of 99.58%, and GCNN achieved and accuracy of 99.98%. It is possible that simpler methods, such as KNN with PCA and multilinear principal component analysis (MPCA), which achieved 71% accuracy, are more suited to complex EEG data than PCA-based dimensionality reduction. Fischer linear discriminant classifier (FLDA) showed an outstanding performance, that is, 97% accuracy, with PSD as the feature extraction method. It shows that FLDA is also an effective method with the right set of features. Graph variational autoencoder (GVAE) and SVM attained 99.78% accuracy showing the robustness of feature learning with ML classifiers. From Table 10, it can be depicted that when it comes to PhysioNet EEG data, more complex approaches with feature-rich algorithms and advanced distance-based procedures tend to far better than simpler ones.

Which authentication algorithm do researchers most frequently employ?

**Figure 13:**
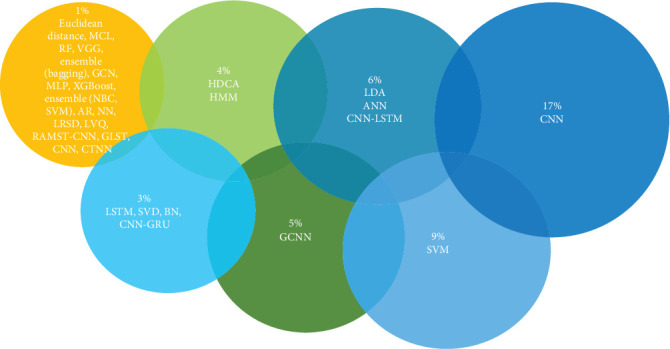
The algorithms used frequently by researchers for EEG-based authentication.

This question is answered by considering two points. The research papers and algorithms used in the last decade were selected to answer this question.  Figure 13 shows the most popular algorithms used over the past decade. DL algorithms, especially CNN, have been widely used by researchers.

### 4.3. Frequently Used Feature Extraction Methods for EEG-Based Authentication

This section helps in finding the answer to Research Question 3: Which feature extraction techniques are mostly used for EEG-based authentication?

Feature extraction is a crucial step in the classification process.  Figure 5 shows some of the most popular feature extraction methods in the literature. It can be observed that AR [95] and PSD [56, 77] are the most common types of feature extraction. The other methods used for feature extraction in the literature are listed in Table 10, including CNN [76], STFT [69], Gaussian filtering [100], the sparse method [72], PCA, maximum scatter difference (MSD) [56], WPD [53], discrete wavelet transform (DWT), LDA [72], and multiple feature extraction techniques [43].

### 4.4. Challenges Addressed in the Literature Regarding EEG-Based Authentication

This subsection helps us find the answer to Research Question 4: How have the challenges in EEG-based authentication been addressed in the past? Which approaches were used?

The inconsistency and variability of the signals caused problems requiring resolution. Wu et al. [72] presented a solution using a multitask approach, eye blinking with cognitive tasks for EEG signal acquisition. Using a multitasking method, they successfully overcame the inconsistency and variability problems of EEG signals with an accuracy of 94.2%. Ozdenizci et al. [97] presented an adversarial network approach to address variability problems. Their method also exhibited good performance with an accuracy of 99.30%. Another problem with EEG-based authentication raised in the literature is the application of forged EEG signals for EEG-based authentication. Therefore, liveness detection was proposed to overcome this problem. To address this problem, Javad et al. [13] proposed cognitive-based EEG signal acquisition. However, their solution did not discuss all aspects of liveness detection, that is, the signal discriminations of an alive and dead person. However, this issue remains a challenge today.

### 4.5. Open Challenges That Need to Be Addressed

This section helps us find answer to Research Question 5: How is the credibility of EEG-based authentication based on recent research on EEG signals? Which open challenges must be addressed?

These questions are answered in three segments.

#### 4.5.1. EEG-Based Authentication Challenges

EEG signals are used successfully for biometric authentication unless fake/forged EEG signals are generated using a GAN [13]. These counterfeit signals are produced on the same lines as the bogus images, and videos are created by acquiring unique features from authentic images and videos. This has resulted in a major setback in the security field of EEG-based authentication. An attacker can invade privacy and breach security if they can generate EEG signals similar to the victims. Attackers can use three possible approaches to create fake EEG signals.

• Copying the victim's brain by thinking or doing the same things as the victim is thinking or doing while EEG signals are being captured.
• Brute force: testing every possible EEG signal as an input. It is measured using entropy. Brute force includes the generation of artificial EEG signals by using GAN [82]. The solution of this issue is a liveness detection method to differentiate between artificial and real EEG signals.
• Sniffing EEG signals of the victim [5].

EEG encryption is one solution to these problems. EEG signals can be easily sniffed using an over-the-air (OTA) transmission protocol. These threats invade privacy and breach security. Recent research has demonstrated the use of EEG signals for the prediction of recently viewed images [27], brainwaves can be translated into text, personal information can be obtained without the knowledge of the person [102], and emotions can also be inferred [103]. Implementing EEG in real-time biometric authentication is challenging and has several limitations. Moreover, universality, permanency, uniqueness, collectability, and user privacy are open challenges that require further research.

#### 4.5.2. Liveness Detection

Differentiation between real and fake EEG signals using liveness detection has been proposed to protect systems from malicious attacks and prevent security breaches [27]. Efforts have been made to detect liveness using biometrics [104]. Some studies have been conducted on liveness detection using images and videos [105]. In a study conducted in 2021, the authors proposed a method for liveness detection. This study has some limitations. They did not cover all the aspects of liveness detection. Moreover, no biomarkers were used to detect liveness. They suggested converting data from 1D to 2D for better liveness detection and suggested using a larger dataset in the future [13]. A study in 1875 found that animal brains produced signals after death; however, these signals were weaker than those produced when a person was alive or dying. This experiment was performed on animals [28]. In recent research, some doctors conducted a similar investigation on humans for the first time in the history of the world and found that the brain remains alive after the death of a person for a few hours. They also captured brain signals when a person was clinically dead [25]. It shook the core of EEG-based authentication because the main reason behind EEG-based authentication is its unavailability after a person's death. Finding a liveness detection method that can address both problems simultaneously, that is, fake EEG signals versus real EEG signals and alive versus EEG signals of a dead person, is crucial. Biometrics other than EEG-based authentication methods have successfully passed the liveness detection test using ECG as a multimodal approach [104].

#### 4.5.3. Proposed Liveness Detection Approach

##### 4.5.3.1. These Aspects of Liveness Detection Must Be Considered

• The drowsiness state of people should be checked to determine if they are drunk; subsequently, they should not be authenticated. Therefore, drowsiness must be detected.
• The conscious level of a person should be checked.
• It should be found that the person is alive or dead.
• The EEG signals used for authentication should be found to be fake or real.

### 4.6. Open Challenges That Need to Be Addressed

This section helps us find answer to Research Question 6. What are the future areas for EEG-based authentication? In the future, EEG-based authentication can be used in diverse areas, some of which are discussed here.

• It can be used in high-security domains, such as the military, high-intelligence areas, and agencies.
• This method can be used to implement multilevel security systems.
• Banks can use EEG-based authentication before conducting transactions. Therefore, an attacker cannot force a person to use their bank account without consent.
• It can be used in educational systems to log into the accounts of both students and teachers. The concentration levels of students can be checked using BCI systems in class.
• Authentication is required before users log into a BCI system. EEG-based authentication can also be used there.
• This method can be used to validate doctors in the field of medicine. Their presence can be ensured by using a doctor's online support.
• When people register with government agencies and are added to a database, it can be used as a biometric. Typically, biometrics involves the use of fingerprints. It is also possible to use multimodal registration, which integrates fingerprints and EEG signals.
• It can be used to register students, lawyers, and practitioners before logging into a system to ensure data reliability and security.
• EEG-based authentication can also be used for gaming purposes. Therefore, the presence of gamers can be ensured before participating in game competitions.

It can also be integrated with smart homes, that is, for opening and locking doors and switching lights on or off. In short, before controlling the home using brainwaves, a person must be authenticated before logging into the BCI application. A more detailed description is given in the Discussion section.

## 5. Discussion

This section presents an analysis of existing difficulties, along with the corresponding techniques for mitigating them, and outlines potential future approaches.

### 5.1. Comprehensive Analysis

This study provides an in-depth study of EEG-based authentication, in which the tools and methodologies used are scrutinized, and the most promising approaches are identified. Several research questions have been formulated to uncover innovative insights from the data. This paper examines the use of EEG-based authentication, its importance, challenges, and potential avenues for future study and application based on an analysis of academic publications published last decade. This paper examines the various disciplines, application areas, and classification methods in the past and the present.

EEG-based authentication is primarily used in mobile technologies [36] and BCI systems [35]. Other areas include the introduction of isolation forests and local outlier factor classifiers, fast EEG-based biometrics for person verification, and real-time data implementation. It has also been used to conduct experiments in new dimensions, such as the effect of auditory stimuli and the IoT [8]. EEG-based identification can be done regardless of the task being performed with an accuracy of 99.3% [69]. Multitask EEG-based authentication is found robust against the instability of signals with an accuracy of 94.2% [72]. The shallow classification and DL approaches exhibited exceptional performance, with the ML algorithm achieving a performance range of 75%–100%. In contrast, DL systems have superior accuracy outcomes, ranging from 96% to 100%. The most frequently used algorithm by the researchers is CNN, and feature extraction often makes use of principal component analysis (PCA) [56, 77] and AR [95]. Most of the issues encountered by both present and historical researchers stem from the inconsistency and variety of the EEG signals. The method proposed by Wu et al. [72] utilized a multitasking strategy, resulting in an accuracy rate of 94.2%. Ozdenizci et al. [97] addressed the problem of variability by using an adversarial network and attained an accuracy of 99.30%.

The use of EEG-based authentication is experiencing a growing presence in the domain of scholarly inquiry. EEG signals are generally recognized for their remarkable resilience compared to other biometric modalities, mostly owing to the conventional notion that EEG signals are incapable of generating after death [5, 21]. The analysis and use of EEG data have received significant recognition within the scientific and medical spheres. Alternative biometric modalities encounter two significant challenges in their implementation. The first challenge pertains to the prevalence of counterfeit images generated by GAI, which undermines the reliability and accuracy of these modalities. The second challenge is the potential misuse of biometric data posthumously, raising ethical concerns around privacy and consent [22]. A study suggested the use of a “liveness detection” technique as a viable alternative for addressing the issue of counterfeit EEG data [23, 24]. The liveness detection technique outlined in the extant literature is specially tailored to address the identification of fabricated EEG data. Nevertheless, this approach fails to address the associated issue of using EEG signals obtained from the brain of a dead person. To address this problem, Javad et al. [13] proposed cognitive-based EEG signal acquisition. However, their solution did not discuss all aspects of liveness detection.

During the period of liveness detection for the notion of EEG-based authentication, it was generally thought that the acquisition of EEG signals from deceased individuals was unattainable. In February 2022, EEG signals emanating from an individual who had died were successfully captured, representing a significant landmark in the chronicles of human civilization [25]. To address this problem, Javad et al. [13] proposed cognitive-based EEG signal acquisition. However, their solution did not discuss all aspects of liveness detection. EEG-based verification relies on two fundamental principles: the inherent inability to counterfeit EEG signals and the impracticability of acquiring EEG data from a dead person. According to recent scholarly investigations, it has been shown that it is possible to collect the EEG signals of a deceased individual. Furthermore, these studies have demonstrated that it is feasible to manipulate and deceive EEG signals, hence rendering the authentication method based on EEG worthless. This work underscores the need to maintain the integrity of all liveness detection components to ensure EEG-based authentication security. Otherwise, the potential risks associated with exploiting biometric systems would likely persist for EEG signals.

#### 5.1.1. Proposed Liveness Detection Approach

Liveness detection for EEG-based authentication should cover the definition provided by ISO in general and these aspects.

• Individual sleepiness levels should be determined; those who seem to be intoxicated should not be authenticated.
• The conscious level of a person should be determined.
• It should be determined that a person is alive or dead whose EEG signals are used.
• It should be found that the EEG signals used for authentication are fake or real.

#### 5.1.2. Suggestions

The problem of liveness detection can be solved by following one of these suggestions.

• Using a multitask authentication method, eye blinking or facial expressions are used to deal with the instability issue of EEG signals [72].
• By identifying biomarkers in the EEG signals, these biomarkers can also be used for authentication.
• Multimodal fusion is widely used in literature [106]. It can also be employed for EEG-based person authentication by using biomarkers with EEG signals (multimodal authentication). ECG-derived respiration (EDR) or ECG can be used along with EEG signals for liveness detection.
• Researchers can utilize GAN to create fake EEG images after converting EEG signals into images. Subsequently, they can distinguish between bogus and real images using image classification algorithms. Much work has been done to differentiate between real and fake images [107].

These well-known and promising feature extraction and classification algorithms, such as PSD [56], PCA, CNN-GRU, and CNN-LSTM [77], can solve current challenges. EEG-based authentication is the future of BCI, IoT, and information security. Therefore, several domains can be merged into a single entity for a solid solution.

#### 5.1.3. Limitation

Further investigation is required to examine the proposals mentioned using experimental procedures thoroughly. In this study, experimental analysis was not conducted on the EEG signals of dead individuals. However, future investigations will use deceased individuals' EEG data to demonstrate their susceptibility to potential risks. This study primarily proposes potential strategies for addressing the challenge of liveness identification. In subsequent applications, the mentioned ideas will be used for liveness detection. These recommendations have significant value for anyone seeking to engage in the development of EEG-based liveness detection techniques.

### 5.2. Future Directions

These are the potential future directions of EEG-based authentication.

#### 5.2.1. Healthcare

The healthcare industry has significant opportunities to implement EEG-based authentication, particularly in patient care, data security, and advanced medical research domains. EEG-based authentication verifies a patient's identification and facilitates the establishment of appropriate associations between healthcare institutions and their respective patients. Additionally, it may contribute to mitigating medical mistakes and effectively managing sensitive patient data. Additionally, it may be used to facilitate entry into critical places and safeguard patient data, hence restricting access to authorized personnel. Additionally, this technology may be used to monitor individuals with intellectual disabilities and the brain activity of those without any cognitive impairments. Consequently, healthcare personnel can enhance their decision-making for patients by considering their mental well-being. This approach has been previously employed in the context of clinical research. EEG-based authentication offers the potential for healthcare services to be delivered with heightened customization and engagement. Specifically, with BCIs, patients can actively interact with medical equipment. Individuals with physical limitations may effectively use medical devices with the implementation of BCI technology. This technology serves the purpose of validating the identities of both patients and healthcare professionals during virtual visits.

#### 5.2.2. Finances

EEG-based authentication in the banking industry offers enhanced security compared to traditional systems while concurrently improving the user experience. This technology enhances the level of security in financial transactions, mitigating the weaknesses often seen in conventional security systems, such as password theft and spoofing. Hence, it is a viable choice for safeguarding financial information. In addition, it can be used inside banking institutions in conjunction with a pair of keys. The use of continuous monitoring of EEG signals offers an enhanced approach to the detection of suspicious behaviors, hence enhancing the robustness of the system. Furthermore, this technology may be used inside airport settings to discern the presence of individuals deemed questionable. This technology offers a user-friendly approach compared to other security measures, allowing users to access their accounts via cognitive processes, eliminating the need for tokens or passwords. To enhance security, implementing multifactor authentication may include using passwords in conjunction with brainwave patterns. Additionally, it can be used to ensure the secure utilization of automated teller machines (ATMs). EEG-based authentication can be integrated into identity verification protocols, such as know your customer (KYC) procedures, used to initiate new accounts or conduct substantial financial transactions. The use of EEG-based authentication has the potential to significantly improve the security and ease of financial transactions and access to accounts.

#### 5.2.3. Education

EEG-based authentication can facilitate access to online educational platforms, therefore ensuring that only duly authorized students are granted entry to educational materials. Additionally, it may be used to detect instances of academic dishonesty in the context of online examinations. Additionally, this technology can monitor children's cognitive states in real time, enabling the assessment of their comprehension levels. Furthermore, virtual reality may be used to deliver specialized instruction to pupils who have cognitive impairments, as well as to conduct targeted training programs. EEG-based BCIs are beneficial in facilitating the interaction between students with movement impairments and their surroundings and promoting engagement with educational materials. It can help enhance comprehension of successful learning strategies used by high-achieving students, hence facilitating the adoption of these strategies by others seeking to optimize their learning outcomes. The curriculum developers may use this data to include instructional information that promotes the development of students' mental and cognitive capacities and their ability to concentrate rather than imposing an excessive workload. Hence, this technological advancement can be used by both instructors and learners alike.

#### 5.2.4. E-Commerce

EEG-based authentication exhibits significant promise within E-commerce, as it can enhance security measures and elevate consumer satisfaction. EEG-based authentication facilitates the verification of a user's identification before granting them access to accounts and sensitive information. Hence, the prevention of fraud is possible. If there are any atypical EEG signals, the ability to regain access may be restored. Instead of relying on conventional password-based authentication or personal information, an alternative approach involves using brain signals to access, mitigating the danger of theft. EEG-based authentication and BCIs enable the monitoring of consumers' cognitive reactions and mental states, hence facilitating the enhancement of their purchasing experience via this data. EEG-based authentication might be valuable for user account recovery and enhancing secure payment processes. This strategy offers a higher level of data protection compared to traditional approaches. Therefore, this aids in establishing trust with the user. Technology can significantly transform the E-commerce sector via the augmentation of security measures, improvement of user experience, and mitigation of fraudulent risks associated with online buying.

#### 5.2.5. Law and Order

EEG-based authentication has significant potential in aiding the resolution of criminal cases via the use of EEG-based authentication and BCIs to monitor individuals' cognitive states. Enhanced security measures at police stations may effectively restrict access to critical information only to authorized persons. Moreover, it may serve as substantiation in legal processes to guarantee the veracity of the assertions. Furthermore, it may be used at various checkpoints to authenticate the identity of immigrants. EEG-based authentication can potentially resolve situations involving unidentifiable people or missing persons. EEG-based authentication may effectively strengthen security and safety measures, offering potential applications in the domains of public safety and justice.

#### 5.2.6. Entertainment Industry

EEG-based authentication in the entertainment business presents several novel applications that enhance user experiences, bolster security measures, and provide fresh avenues for interactive entertainment. Using EEG-based authentication and BCIs makes monitoring users' cognitive reactions possible. This monitoring enables the provision of enhanced entertainment experiences according to user's preferences, which are decided by their mental states. EEG-based authentication has the potential to be used in several domains, including video games, enhanced virtual reality experiences, virtual concerts, virtual reality tours, gaming tournaments, content production, and other related areas.

### 5.3. Ethical Concerns

When it comes to using EEG devices for extended periods of time, the most prevalent issue is safety. It is stated by the authors that the usage of these devices might have the potential to cause permanent negative effects and hazards, particularly when they are used by youngsters who are still growing. However, there has been no discovery of evidence as of yet. There should be further investigation done on this. Additionally, there are certain nonmedical concerns that may arise, such as the fact that intensive training and cognitive attention may result in frustration and may place a load on the individual on a physical, emotional, and financial level [108]. When someone becomes unduly dependent on EEG equipment, the failure of the device may put the individual's life in peril, as is the situation when the individual is attempting to operate a wheelchair and it fails in the middle of a busy road [109]. EEG devices, which are capable of reading the mind, may be a significant risk to both individuals' privacy and their ability to connect with others. There are significant questions about identification that are brought up by the intrusive BCIs. Among them are changes in behavior that might lead to impulsiveness, mania, and gambling [110]. There have been reports from some patients that they feel as if they are under control or as if they are an electric doll [111]. According to Zehr, technologies that have the potential to increase human intellect and physiology will also have the ultimate effect of transforming humans [112]. Researchers have to discover a means to strike a balance between the possible dangers and future breakthroughs in technology, taking into consideration the ethical problems that are involved.

## 6. Conclusion

The current study provides a comprehensive overview that elucidates key findings and perspectives within the field of EEG-based authentication. The results of this study may provide valuable insights for scholars and professionals in discerning effective approaches, existing technological advancements, and prospective advancements in EEG-based authentication. Moreover, this paper examines the use of EEG-based authentication in light of the research papers published in the last decade. It explores this technology's importance, its challenges, and potential avenues for future development. This study investigated the breadth of the area, the historical and contemporary classification methods, and the potential future trajectories for EEG-based authentication. The current study presents the suggestions and results derived from the review conducted. EEG-based verification relies on two fundamental principles: the inherent inability to counterfeit EEG signals and the impracticability of acquiring EEG data from a dead person. According to recent scholarly investigations, it has been shown that it is possible to acquire EEG signals from individuals who have passed away.

Furthermore, these studies have demonstrated that it is feasible to deceive EEG-based authentication systems by falsifying EEG signals. Consequently, the effectiveness of authentication methods relying on EEG signals is questioned. This work highlights the critical need to preserve all aspects of liveness detection to maintain the security of EEG-based identification. Otherwise, as shown in earlier instances, the potential risks associated with exploiting biometrics will persist.

This study highlights the significance of using EEG-based person authentication as a dependable and safe biometric method. The claim acknowledges the inherent benefits of EEG in comparison to other biometric approaches, including its resistance to fabrication and its ability to use postmortem data. Nevertheless, it also underscores the growing complexities associated with EEG-based verification, including the requirement for robust “liveness detection” techniques to counteract the potential risks posed by counterfeit EEG signals. The research offers a comprehensive examination of previous and current endeavors and proposes an updated approach for detecting liveness in EEG-based authentication. The suggested technique enhances the integrity of EEG-based authentication and paves the way for future study and advancement in this domain. The ongoing progress of EEG technology necessitates the constant vigilance of researchers and practitioners in adapting and enhancing authentication techniques to maintain their efficacy in practical scenarios. The prospective research applications of EEG-based authentication provide promising opportunities for augmenting security and user verification across several fields. This paper offers a significant addition to the existing academic discussion on EEG-based person authentication, promoting further investigation and advancement in this area of research. Given the latest discoveries in the field of EEG signals, it is essential to include comprehensive liveness detection measures in EEG-based authentication systems to maintain such authentication methods' resilience.

Further research is required to get EEG data from individuals' postmortem. This research investigation has revealed previously unaddressed research gaps in existing literature. In addition to identifying research gaps, this study proposes potential answers and outlines future possibilities, providing valuable guidance for other researchers.

## Figures and Tables

**Table 1:** List of abbreviations and their explanation.

**Abbreviation**	**Explanation**
RFE	Recursive feature elimination
LMS	Least mean square
ICA	Independent component analysis
CCA	Canonical correlation analysis
PSD	Power spectral density
EMD	Empirical mode decomposition
IMF	Intrinsic mode functions
AR	Autoregressive
SMOTE	Synthetic minority oversampling technique
STFT	Short-time Fourier transform
FIR	Finite impulse response
MCL	Mean curve length
KFD	Katz fractal dimension
PSD	Power spectral density
FC	Functional connectivity
PCA	Principal component analysis
MPCA	Multilinear principal component analysis
KNN	*K* nearest neighbor
RHO	Synchronization index measures deviation of relative phase
PLV	Phase locking value
COH	Spectral coherence connectivity
FLDA	Fischer linear discriminant classifier
GCNN	Graph convolutional neural network
GVAE	Graph variational autoencoder
SVM	Support vector machine
ML	Machine learning
DL	Deep learning
LDA	Linear discriminant analysis
CRR	Correct recognition rate
ED	Euclidean distance
DEAP	Database for emotion analysis using physiological signals
SVDD	Support vector data description
EER	Equal error rate
FAR	False acceptance rate
FRR	False rejection rate
LVQ-NN	Learning vector quantization neural network
MSC	Magnitude squared coherence
EB	Eigenbrain
ETB	Eigentensorbrain
HTER	Half total error rate
EEG	Electroencephalography
BCI	Brain-computer interfaces
GAN	Generative adversarial network
ISO	International Standards Organization
IEEG	Intracranial electroencephalography
MEG	Magnetoencephalography
SEEG	Stereoelectroencephalography
CNS	Central nervous system
ERP	Evoked-related potential
VEP	Visual evoked potential
SSVEP	Steady-state evoked potential
MLP	Multilayer perceptron
LDA	Linear discriminant analysis
NBC	Naïve Bayes classifier
VGG	Visual geometry group
NN	Neural network
DWT	Discrete wavelet transform
WPD	Wavelet packet decomposition
MSD	Maximum scatter difference
RNN	Recurrent neural network
LSTM	Long short-term memory
BLSTM-NN	Bidirectional long short-term memory with neural network
RAMST-CNN	Residual and multiscale spatiotemporal convolutional neural network
GSLT-CNN	Global spatial and local temporal convolutional neural network
CTTNN	Convolutional tensor train neural network
GRUs	Gated recurrent units
SLR	Systematic literature review
ICA	Independent component analysis
XGBoost	Extreme gradient boost
GAI	Generative artificial intelligence
RF	Random forest
HMM	Hidden Markov model
ANN	Artificial neural network
LVQ	Linear vector quantization
LRSD	Low-rank sparse decomposition
BN	SD network
GM	Gaussian mixture
HDCA	Hierarchical discriminant component analysis
DFT	Discrete Fourier transform
UCI KDD	University of California, Irvine Knowledge Discovery Database

**Table 2:** Articles published in the last 10 years on EEG-based authentication.

**Publisher**	**Time frame**	**No. of occurrences**	**Reference**
IEEE	2014–2023	128	[14]
MDPI	2014–2023	25	[16]
PubMed	2014–2023	76	[15]
ACM	2014–2023	3119	[20]
Springer	2014–2023	10	[19]
ScienceDirect	2014–2023	718	[18]
Tylor & Francis	2014–2023	69	[17]

**Table 3:** Applications of EEG-based authentication.

**Domain, authors (year)**	**Study area**	**Dataset**	**EEG channels**	**Sampling rate**	**Methods/algorithms**	**Result**
Mobile EEG–based biometric authentication (Kjeldgaard et al.) 2012 [38]	The uniqueness of short EEG recordings	Self-developed	P7, P8, O1, O2	Null	PSD and wavelet analysis. RESTful web interface	EEG can be used with mobiles for biometric authentication of a person
Brain-computer interface (Lin et al.) (October 2017) [39]	Brain-computer interface and ERP + DS fusion	Self-developed	FP1, FP2, Pz, Oz	250 Hz	Ensemble of NBC, SVM	Application of DS fusion improved classification from 60% to 75%
Brain-computer interface in IoT (Ramzan and Shidlovskiy) 2018 [8]	Security breach of old security methods + importance of EEG	Null	Null	Null	Review based	Rely totally on EEG and EMG signals of brain-computing interface
Cryptography and biometrics (Nguyen et al.) 2019 [12]	EEG-based cryptography key generation	From the University of California & Graz IIIa from BCI competition 2005	Random EEG features	256 Hz, 250 Hz	AR spectral analysis with Burg's method	Longer keys generation, using random EEG features More robust keys can be generated by using EEG
EEG-based authentication (Barayeu et al.) 2020 [40]	Usage of VGG + deep NN	Self-developed, 105 subjects	8, 16, and 64 channels	160 Hz	VGG-NN-SVM, decoder, PCA-SVM decoder	88, 87, 90% 92, 92, 95%
EEG-based authentication (Cooper et al.) 2021 [37]	Binary classification	PhysioNet	64 channels	160 Hz filtered to resample at 12–14 Hz	ICA and NN	100%
EEG-based authentication (Yang, Libert, and Van Hulle) 2021 [41]	Real-time implementation	Self-developed	8 channels	100 Hz	Bagging with LSTM	92.5%
EEG-based biometric for identification and authentication (Yahyaei) 2022 [42]	Fast EEG-based biometric via mean curve length (a new feature)	PhysioNet	64 channels	160 Hz	MCL	95%
EEG-based human identification (Tian et al.) 2022 [43]	Using multiple features	PhysioNet	64 channels	160 Hz	GCNN	98.56%
EEG-based authentication (Alzahab et al.) 2022 [44]	Effect of auditory stimuli	Local, and PhysioNet	T7, F8, Cz, P4	200 Hz	MLP, KNN, XGBoost.	EEG is genre-independent (auditory stimuli did not affect EEG)
EEG-based authentication. (Luis et al.) 2023 [45]	Introduction of isolation forest and local outlier factor classifiers	Self-developed	15 channels	256 Hz	Isolation forest and RF classifiers	Accuracy of 82.3%, a precision of 91.1%, and a recall of 75.3%

**Table 4:** Data collection methods used in the literature.

**Ref**	**Data collection method**	**Strength**	**Weakness**	**Solution**
[38]	EMOTIV EPOC headset with 14 electrodes	Emotive headsets have low cost Aids in real-time data collection	Low signal quality may lead to noisier signals	Headsets with minimum noise should be used

[39], [41]	Dry-contact EEG sensors	There is no need for conductive gel Maintain good contact with the scalp, even with hairs The wireless setup makes mobility easy	Lower signal quality as compared to wet electrodes Inclined to motion artifacts	Apply adaptive filtering techniques or use ICA

[40]	64-channel EEG system	It provides detailed spatial information that helps in obtaining the specific brain patterns of a person It provides large dataset for training	It needs extensive setup Time-consuming	Fewer channels can make the system more practical

[12]	1st dataset: Collected from the University of California, Irvine (UCI) Knowledge Discovery in Databases (KDD) Archive. Each subject is shown visual stimuli 2nd dataset: Data was recorded with a 60-channel EEG amplifier	1st dataset (alcoholism dataset) has 122 subjects, which introduces statistical stability Task-specific datasets, that is, use of visual stimuli or motor imagery	2nd dataset is very small Both datasets are recorded under controlled conditions that does not reflect the conditions of real world Experiments done by using these datasets cannot be used for the real-world application	Use portable lower channel EEG devices Use data augmentation techniques for smaller datasets

[37] [43]	EEG motor movement/imagery dataset from PhysioNet: Dataset has 109 subjects each performing some motor movements	With 109 samples, there is lot of data for training High temporal resolution	As the dataset is recorded in a controlled environment, so there is limited real-world applicability High channel count	Add data augmentation techniques, that is, adding simulated real-world noise

[42]	Gel-based disk electrodes	High signal quality Detailed reliable data Stability	Long setup time User discomfort Limited portability	Use of wearable EEG devices Use advanced electrode design that can provide balance in signal quality and portability

[45]	Data is collected using EMOTIV EPOC + V1.1 wireless headset	Ease of setup Adequate spatial coverage	Low signal quality as compared to gel-based system Sensitive to motion artifacts	Use filtering techniques to removing artifacts and noise Consider semidry electrodes to improve signal quality

**Table 5:** Preprocessing methods used in literature.

**Ref**	**Preprocessing method**	**Strength**	**Weakness**	**Mitigation strategies**
[38]	Butterworth method to extract signals between 0.5 and 40 Hz	Butterworth helps in removing unwanted signals Minimal signal distortion	It may remove high-frequency components that may contain a unique feature	Use adaptive filtering, that is, LMS. Useful in real-world scenarios ICA: It enhances signal quality and removes artifacts

[39]	CCA	It is very effective for removing noise. Enhances signal quality	Computationally expensive	Use ICA with artifact classification

[40]	Sliding windows are used for segmented EEG signals Filtered with zero phase delay Bandpass filter, 1–50 Hz is used	Bandpass filter isolates the frequency range relevant to motor and cognitive signals Sliding window creates multiple trails for each session	Sliding windows with 75% overlap may introduce redundancy Overlapping of signals introduces complexity	Reduce the overlap in a sliding window Use of batch processing can reduce computational cost

[12]	Frequency subbanding	Focus on the relevant information, which enhances reliability Noise reduction by using relevant frequency bands	It is very important to choose the correct frequency range, which varies across persons, so it is very difficult to generalize	Optimize frequency band selection, by using adaptive algorithms. These algorithms can dynamically select frequency bands based on individual profiles Use PCA for dimensionality reduction

[37]	Frequency filtering, beta wave range is isolated from rest of the bands, which contains most relevant active state information	Noise reduction, as only a specific frequency band is chosen Beta frequency band focuses on motor activity thus is most relevant to authentication task	Distortion at signal's boundaries Artifact residue	Use wavelet-based filters, as they are better in preserving signal's structure

[41]	Butterworth bandpass filtering, a fourth-order Butterworth filter with cutoff frequency 1–30 Hz is used STFT is used to find spectrograms	Butterworth filters simply isolate desired frequency bands. It also provides smooth frequency response Downsampling reduces data size and thus lowers computational load	Some useful information outside the cutoff frequency is ignored	Use wavelet decomposition that can focus on multiple frequency bands

[42]	Low-pass filtering, FIR filter with cutoff frequency 50 Hz Segmentation	Low-pass filter removes high-frequency noise including power line interference By cutting off high frequency, the study emphasizes lower frequency bands, that is, delta, then alpha and beta Segmentation improved data volume	Low-pass filter can introduce edge artifacts at the beginning and end of filtered signals Fixed segmentation can result in the loss of long-range temporal information	Use of wavelet-based filtering could retain a broader range of frequency Overlapping signal segments before filtering can reduce artifacts Implement variable segment lengths

[43]	Sliding window segmentation	Overlapping segments increase the number of data samples Captures temporal patterns	Risk of artifact and noise	Remove artifacts and noise

[45]	Signal segmentation	Increases data volume Captures temporal patterns	Loss of long-term information Introduces boundary artifacts	Use overlapping windows Choose adaptive segmentation

**Table 6:** Feature extraction methods used in the literature.

**Ref**	**Feature extraction method**	**Strength**	**Weakness**	**Solution**
[38]	PSD Wavelet analysis	Provides a unique set of features Provides high-resolution temporal and frequency information Provides individualized patterns in brainwaves	High dimensionality Sensitive to noise High computational cost	Use PCA to reduce dimensionality Use LDA, it will help in dimensionality reduction Feature selection techniques, that is, RFE windowed signal processing

[39]	PSD, it is used to analyze power across different frequency bands	Provides a clear picture of brain signals, allowing the identification of various features in frequency bands	It is sensitive to noise	Use noise suppression techniques Also apply PCA

[40]	EMD is used to decompose EEG signals into IMFs. PSD, Shannon entropy, log entropy, and sample entropy are calculated for each IMF	EMD captures frequency-specific information Entropy features capture the complexity of EEG signals	High dimensionality Computationally intensive	Use PCA to reduce high dimensionality Selecting fewer IMFs can also reduce complexity

[12]	AR spectral analysis with Burg's method	AR provides high resolution and smoothness In comparison with PSD, Burg's method provides smoother power spectrum Burg's method avoids bias in autocorrection	Burg's method is computationally more expensive than PSD	Apply efficient estimation techniques Simplify the AR process

[37]	ICA, it converts EEG signals into independent components SMOTE addresses class imbalance problem by creating synthetic data	ICA enhances signal interpretation Data dimensionality reduction can be achieved by using ICA SMOTE reduces class imbalance problem	ICA depends on the high-quality data Computationally expensive SMOTE: Risk of overfitting	Use Fast_ICA Ensure data collection in controlled environment Combine SMOTE with undersampling

[41]	STFT, to generate spectrograms. Spectrograms provide time-frequency representation of EEG signals	It captures detailed spectral information, which helps in identifying power and distribution of various brainwaves over time It is easy to compute and is well supported by many software tools It can be easily used in real-world scenarios	It uses a fixed window size A smaller window provides better time resolution but poor frequency resolution Computational complexity	Use wavelet transform instead of STFT, where different frequency bands are analyzed with varying time windows Use parallel processing techniques to reduce computational complexity

[42]	MCL, it computes complexity of EEG signal by calculating the mean of absolute values of the first-order finite difference (discrete derivative) KFD is used to measure the fractal dimension of EEG signal PSD measures the power distribution across different frequency bands	MCL is computationally efficient and also captures the complexity of EEG signals KFD captures fractal natures of EEG signals. It is also scale invariant PSD provides detailed view of power distribution	MCL is sensitive to noise KFD can vary based on chosen sampling rate, and it is also computationally complex which can make it difficult to use in real-time scenarios PSD is sensitive to artifacts	For MCL, apply Wavelet denoising before calculating MCL. For KFD, standardized sampling rate. Use simplified fractal dimension measures, that is, Higuchi's method. For PSD, use artifact removal techniques

[43]	Common FC features, that is, Pearson's correlation coefficient, COH, PLV, mutual information	Pearson's correlation coefficient measures the linear correlation between two signals COH captures frequency-domain relationships PLV measures phase synchronization, which is used to find time-domain relationships between signals. It is invariant to the amplitude of the signal	Pearson's correlation coefficient and coherence capture only linear dependencies. It may miss complex or nonlinear EEG signals PLV is sensitive to noise	Use a combination of linear and nonlinear features For PLV use noise reduction techniques, that is, adaptive filtering or wavelet denoising

[45]	Wavelet-based statistical features	Wavelet decomposition captures both time and frequency-domain information. That is how meaningful information across various frequency bands from nonstationary EEG signals can be captured Robustness to noise	Computationally expensive Risk of overfitting Sensitive to artifacts	Use dimensionality reduction methods, that is, PCA Use artifact-removing preprocessing methods

**Table 7:** Highlights of algorithms used for classification in EEG-based authentication, including validation, feature extraction methodologies, and results obtained.

**Ref**	**Dataset**	**Data split method**	**Feature extraction method**	**Parameters**	**Classifier name**	**Performance metric**	**Result**
[62]	The private dataset collected by Neurosky MindWave headset	Randomized approach	Levinson–Durbin and Burg algorithms	NUL	LDA	CRR = 96.7%	As the number of test samples increased, the system's CRR improved

[61]	Private dataset from 17 subjects	10-fold cross-validation	PSD	NUL	ED, LDA, SVM	Single run, ED = 99%, LDA = 99%, SVM = 99% Crosstime, ED, LDA SVM = 80%	Classifiers performed comparably well for single-run data

[70]	A private dataset of 10 subjects was recorded using 18 electrodes according to the International 10–20 system	10-fold cross-validation	Pointwise biserial correlation for temporal feature selection and least squares estimation for dynamic features	NUL	SVM	Accuracy = 86.1%, FAR = 13.9%, FRR = 13.9%	The same values of FRR and FAR mean the system is equally likely to incorrectly accept an imposter or reject a genuine user

[72]	A private dataset with 40 subjects	Open and closed sets are used. 1350 samples are used for training in a closed set, and 150 samples are used for testing. 1000 samples are used in a closed set for testing	By averaging the EEG responses, ERP features are calculated For eye-blinking signals, time-domain morphological features were extracted	Training parameters: The learning rate is set to 1 Batch size = 100 Epochs = 50	CNN	Closed set result: Accuracy = 97.6%, FAR = 2.71%, FRR = 2.09% Open set result: FAR = 3.9% Permanence result: FRR = 3.87%	This research study shows the effectiveness of combining EEG and eye-blinking features

[71]	A private dataset of 4 subjects (A, B, C, and D) and an additional 75 subjects	50 feature vectors for training and 75 feature vectors for testing	EEG alpha band is extracted. The 10th-order AR model is used to extract features from the alpha band	NUL	LVQ-NN	A: 80% B: 77.8% C: 84.4% D: 80%	Results affirm EEG-based person authentication, with individual accuracy rates between 77.8% and 84.4%

[65]	EEG data from the PhysioNet database with 109 subjects	Each 1-min EEG recording is divided into 6 parts; 5 parts are used for training, and 1 part is used for testing	MSC	NUL	KNN	64 channels, accuracy = 100% 10 channels, accuracy = 100% 6 channels, accuracy = 97.24% 5 channels, accuracy = 95.4%	This research demonstrates that decreasing the number of channels from 64 to 10 did not impact the accuracy

[73]	EEG dataset collected from 30 subjects	EEG data is divided into 5-min frames. 59 frames are used for training, and 39 frames are used for testing	PCA and MPCA are used to extract EB and ETB	NUL	LDA	EB + LDA = 83.2% ETB + LDA = 87.9%	Results show that ETB combined with LDA provides better results

[74]	EEG dataset from PhysioNet database	*k*-fold cross-validation is applied on 109 subjects such that, for each subject, five epochs were used for training and 1 epoch was used for testing	PSD	NUL	FLDA	Eyes closed, accuracy = 96.1%–98.9% Eyes open, accuracy = 95.3%–97.2%	Results showed high accuracy for biometric identification, particularly in the eyes-closed condition

[75]	X2 RSVP, CT2WS RSVP, XB driving, DEAP emotion Total 157 subjects	90% of epochs from each session are used for training and 10% for testing	PSD, AR	Learning rate = 0.001 Momentum = 0.9 Iteration steps = 50,000 Momentum-based optimizer	GSLT-CNN	Accuracies X2 RSVP = 99%, CT2WS RSVP = 93%, XB driving = 97% DEAP emotion = 99% Combined dataset = 96%	The GSLT-CNN outperformed shallow classifiers

[76]	MAHNOB-HCI dataset	90% used for training,10% reserved for testing	Features from CNN's fully connected layer are extracted	Learning rate = 0.001 Batch size = 128 samples Epochs = 10 Activation function in fully connected layer = softmax	CNN-SVM	99.87%	The results show that emotional EEG data with a CNN-SVM model is highly effective for person identification

[35]	EEG data is collected by EMOTIV EPOC + headset Gait data	An eightfold cross-validation split is used	EEG delta band features were extracted using an attention-based encoder–decoder RNN	Learning rate = 0.001 Iterations = 1000	RNN	Accuracy: For EEG data = 99.96% For gait data = 99.61% False acceptance rate = 0% False rejection rate = 1%	DeepKey is a highly effective and secure biometric authentication system. By combining EEG and gait modalities, it achieved near-perfect accuracy

[77]	DEAP dataset	10-fold cross-validation	CNN layers are used for feature extraction	Batch size = 256 Learning rate = 0.003 Models were trained until convergence	CNN-GRU, CNN-LSTM	CRR: CNN_GRU = 100% CNN_LSTM = 99.79%	CNN-GRU offers a reliable and efficient approach for EEG-based person identification

[78]	Multimodal dataset is used, dynamic signatures + EEG signals	80% data is used for training, and 20% data is used for testing	DFT was used to extract Gamma band features	Learning rate = 1e − 4, momentum = 0.9	BLSTM-NN	Accuracy = 98.78%, FAR = 3.75%, HTER = 1.87%	Results indicate that combining EEG and dynamic signature data significantly enhances identification accuracy

[79]	Two datasets are used, PhysioNet and a private dataset	Five-fold cross-validation is used	PSD	Learning rate = 0.001, batch size = 200	GCNN	CRR = 90%–99.98%	The study shows the robustness of GCNN for person's identification

**Table 8:** Algorithms used for EEG-based cryptography for a person's authentication.

**Algorithms**	**Methods**	**Accuracy/key length in bits**	**Dataset**
Key combining EEG-based biometric cryptosystems	Support vector machine and BN [46]	98.46%	BCI Dataset I with 3.05 error rate BCI Dataset II with 4.35% error rate

Key generation EEG-based biometric cryptosystems	Fuzzy extractor [47]	128	10 subjects
	Fuzzy commitment [85]	400, 21	42, 10 subjects
	Quantization [86]	230, 256, 62	120, 3, 10 subjects

Key binding EEG-based biometric cryptosystems	Fuzzy commitment [87]	400	422 subjects 1.87% error rate
	Fuzzy vault [88]	99%	BCI competition 2008

**Table 9:** Summary of the datasets, channels, and findings of different papers.

**Publications**	**Data**			**Processes**		**Output**
**Authors**	**Tasks**	**Dataset/subjects**	**Channels**	**Feature extraction**	**DL/ML/both**	**Findings**
(Salem and Lachiri) 2019 [76]	VEP	MANHOB-HCI/30 subjects	32	CNN	SVM (both)	5-channel EEG data (PO3, PO4, O1, Oz, and O2) filtered in beta frequency has the most discriminative features. Accuracy: 99.9%

(Kong et al.) 2018 [69]	RP, VEP, ERP, MI	BCI Graz dataset/9 subjects	22	STFT	LRMD	EEG-based identification can be done regardless of the task that is being performed. Accuracy: 99.3%

(Seha and Hatzinakos) 2020 [94]	Auditory	40 subjects	7	Gaussian filtering	LDA	It is found that AEP plays an effective role in EEG-based authentication. Even with short EEG recordings, good accuracy is achieved. Accuracy = 94.55% and 96.5%

(Wu et al.) 2018 [72]	Visual evoked. Eye blinking	150 in the first session. 255 in the second session	16	Sparse method	CNN HDCA	An EEG-based person authentication system that used multitasking performed better than the single-task EEG-based authentication. Accuracy: 97.6, FAR = 2.71, FRR = 2.09

(Koike-Akino, Mahajan, and Marks) 2016 [30]	ERP	25	11	PCA and PLS	LDA	Dimensionality reduction plays an effective role in classification. Accuracy 96.70%

(Birgham and Brigham and Kumar) 2010 [95]	Imaginary speech	7 subjects. The self-made dataset is used	128	Univariate AR model	KNN, *K* = 3	Useful electrodes are separated from the ones that are not useful. Accuracy increases when only useful electrodes are used. Accuracy: 0.60–0.72

(Thomas and Vinod) 2018 [56]	No task	109 subjects. Self-made dataset	19	PSD	Crosscorrelation values	The gamma band can be used for feature extraction when the person is not performing any task. By using it, 90% accuracy is achieved

(Bashar et al.) 2019	No task	Null	Null	MSD	ECOC-SVM	Accuracy: 94.44

(Zhang et al.) 2022 [32]	Steady-state visual-evoked potential	70 subjects	64	Discrete cosine transformation	ANN	Reduce signal loss to 50%–81%

(Zeynali et al.) 2019 [96]	Motor imagery	7 subjects	6	DWT	SVDD	Accuracy: 99.90%

(Wu et al.) 2018 [72]	Eye blinking + cog	40 subjects	Null	LDA	HDCA	Multitask EEG-based authentication is found robust against the instability of signals. Accuracy =94.2

(Ozdenizci et al.) 2019 [97]	RSVP	10 subjects	16	PCA	CNN	This study proposes an adversarial inference approach, that is, session invariant. It is found that this method solves the variability issue of EEG signals. Accuracy: 99.30

(Sun, P.-W., and, B. Lo) 2019 [80]	MI	109 subjects	16	Null	1D-CNN + LSTM	This approach outcasts state-of-the-art algorithms, w.r.t accuracy, and EER. Accuracy: 99.58

(Wilaiprasitporn et al.) 2018 [77]	VEP	40 subjects, DEAP dataset	5	PSD	CNN-GRU	It is found that CNN-GRU is better in terms of computational speed therefore can be used for real-time applications. Accuracy: 99.17

**Table 10:** Performance comparison of various classifiers, on PhysioNet.

**Ref**	**Channels**	**Subjects**	**Feature extraction method**	**Classifier**	**Database**	**Accuracy (%)**
[98]	56	108	PSD, COH	Mahalanobis distance–based classifier	PhysioNet	100
[73]	19	30	PCA, MPCA	KNN	PhysioNet	71
[74]	1	109	PSD	FLDA	PhysioNet	97
[99]	64	109	RHO	Mahalanobis distance–based classifier	PhysioNet	98.83
[80]	16	109	Raw EEG	CNN, LSTM	PhysioNet	99.58
[80]	4	109	Raw EEG	CNN, LSTM	PhysioNet	94.28
[79]	64	109	PLV	GCNN	PhysioNet	99.98
[100]	64	109	GVAE	SVM	PhysioNet	99.78
[101]	3	109	Raw EEG	CNN	PhysioNet	98.04

## Data Availability

The authors have nothing to report.
